# Supplementary material for: Classification of the human phox homology (PX) domains based on their phosphoinositide binding specificities
Source: Nat Commun. 2019 Apr 4;10:1528. doi: 10.1038/s41467-019-09355-y (PMC6449406; doi:10.1038/s41467-019-09355-y)
Supplement: Supplementary file 1 — Supplementary Information [file 41467_2019_9355_MOESM1_ESM.pdf]

Supplementary Information

Supplementary Table 1. Previously reported structures and PtdIns*P*-binding specificities of mammalian PX proteins.

Supplementary Table 2. PX domain constructs used in this study.

Supplementary Table 3. Primers used in this study for site-directed mutagenesis of PX domains.

Supplementary Table 4. ITC thermodynamic parameter table.

Supplementary Table 5. Blitz kinetic parameter table.

Supplementary Figure 1. Gel filtration profiles of purified PX domain used in this study. Affinity purified PX domain proteins with GST-tags removed were examined by gel filtration on a Superdex200 10/30 column.

Supplementary Figure 2. PX domain binding to phosphoinositides measured by ITC. Binding of water-soluble phosphoinositide headgroup analogues (500 μM) titrated into selected PX domain proteins (20 μM) and measured by ITC. Top panels show the raw data and bottom panels represent the integrated and normalized data fit with a 1:1 binding model. The binding affinities (K_d) are provided in **Supplementary Table 1**.

Supplementary Figure 3. Comparison of PX domain binding to different phosphoinositides measured by ITC.

Binding of water-soluble phosphoinositide headgroup analogues (500 μM) titrated into selected PX domain proteins (20 μM) and measured by ITC. Top panels show the raw data and bottom panels represent the integrated and normalized data fit with a 1:1 binding model. The binding affinities (K_d) are provided in **Supplementary Table 1**.

Supplementary Figure 4. PX domain binding to phosphoinositides measured by BLItz. Biotinylated PC/PE liposomes containing the indicated phosphoinositides were coupled to a streptavidin probe and binding to PX proteins measured at a single concentration of 20 μM using the BLItz system. Binding kinetics were calculated using Prism software and are provided in **Supplementary Table 2**.

Supplementary Figure 5. Comparison of PX domain binding to different phosphoinositides measured by BLItz.

Biotinylated PC/PE liposomes containing the indicated phosphoinositides were coupled to a streptavidin probe and binding to PX proteins measured at a single concentration of 20 μM using the BLItz system. Binding kinetics were calculated using Prism software and are provided in **Supplementary Table 2**.

Supplementary Figure 6. Crystal structures of the SNX15, SNX23, SGK3, and SNX32 PX domains.

(A) Cartoon representation of the PX domain of SNX15 bound to sulphate (crystal form 1). The protein forms a domain-swapped dimer, whereby the α1 helix is longer than normal leading to swapping of the subsequent α2 and α3 with an adjacent molecule in the crystal lattice. (**B**) Cartoon representation of the PX domain of SNX15 (crystal form 2). The protein still forms a domain-swapped dimer, but the C-terminus of the α1 helix unfolds to form a longer extended structure. (**C**) Analytical gel filtration profile of the SNX15 PX domain (compared with the standard calibration curve). The solid line represents the normalized UV absorbance at 280 nm. The chromatogram peak represents the corresponding measured molecular weight in kDa. (**D**) (Left) Cartoon representation of the SNX23/Kif16B PX domain. (Right) Overlay of the three SNX23 chains within the asymmetric unit with the previous SNX23 PX domain structure in complex with sulphate (PDB ID 2V14). (**E**) (Left) Cartoon representation of the SGK3 PX domain. (Right) Overlay of the SGK3 structure with the two chains of the asymmetric unit within the previous SGK3 PX domain structure (PDB ID 1XTN).

Supplementary Figure 7. Sequence and crystal structure of the SNX32 PX domain in complex with IncE.

(**A**) Sequence alignment of human SNX32, SNX5, SNX6 and SNX1 PX domains. Conserved residues are indicated in red. Side-chains that directly interact with IncE in the crystal structure are indicated with black circles. Alignment was made with ESPRIPT (Robert and Gouet, 2014). (**B**) Cartoon diagram (shown in wall-eye stereo) showing the crystal structure of the human SNX32 PX domain (green) in complex with IncE (residues 108-132) (orange). All structure images were generated using PyMOL (Delano Scientific). The extended helix-turn-helix structure composed of helices α' and α'' extends towards to the top of the image. (**C**) Superposition of the crystal structure of the SNX32 PX domain complex with IncE (green and

orange) with that of the previous SNX5-IncE structure (black and blue) (PDB ID 5TGI) (Paul et al., 2017). Structures are shown in backbone ribbon representation. (**D** and **E**) Two close up views of the SNX32-IncE interface highlighting the important interactions between the two proteins. (**F**) The relative sequence conservation of SNX32 side-chains are plotted on the surface representation from blue (highly conserved) to white (not conserved). The IncE peptide is shown in orange cartoon representation. The conservation was calculated using CONSURF (Ashkenazy et al., 2016).

Supplementary Figure 8. NMR structure of the SNX25 PX domain.

(A) 2D ^{1}H-^{15}N-HSQC spectra of the SNX25 PX domain at 500 μM with sequence-specific assignments shown. **(B)** The 20 lowest energy NMR structures calculated for the SNX25 PX domain, shown in wall-eye stereo. **(C)** Solution NMR structure of the SNX25 PX domain in ribbon diagram. **(D)** Sequence alignment of the SNX25 PX domain with the other human RGS-PX family members. Secondary structure is indicated based on the SNX25 NMR structure. **(E)** Structural overlay of the SNX25 PX domain NMR structure with the SNX14 and SNX19 PX domains determined by X-ray crystallography (PDB IDs 4P2J and 4PQO respectively) (Mas et al., 2014).

Supplementary Figure 9. Conservation of canonical and secondary site amino-acid sequences needed for phosphoinositide binding.

Black and grey indicates non-conservative and conservative substitutions respectively. The phosphoinositide-binding group is indicated in the last column, and lower case grey letters indicate predictions of the phosphoinositide-binding preference based on the sequences of the proteins.

Supplementary Figure 10. Binding of SNX25 to phosphoinositides by NMR.

2D ^{1}H-^{15}N-HSQC spectra of the SNX25 PX domain at 50 μM were recorded in the presence of increasing molar ratios of the indicated di-C8 soluble phosphoinositide species. PtdIns and mono-phosphorylated PtdIns3*P* and PtdIns4*P* did not show significant binding. Di and tri-phosphorylated species however resulted in significant chemical shift perturbations.

Supplementary Figure 11. PX domain mutants binding to phosphoinositides measured by ITC.

Binding of water-soluble phosphoinositide headgroup analogues (500 μM) titrated into selected PX domain proteins and their mutants (20 μM) measured by ITC. Top panels show the raw data and bottom panels represent the integrated and normalized data fit with a 1:1 binding model. The binding affinities (K_d) are provided in **Supplementary Table 1**.

Supplementary Figure 12. Raw gel images for Figure 1 and Figure 2.
All gels were stained with Coomassie Blue.

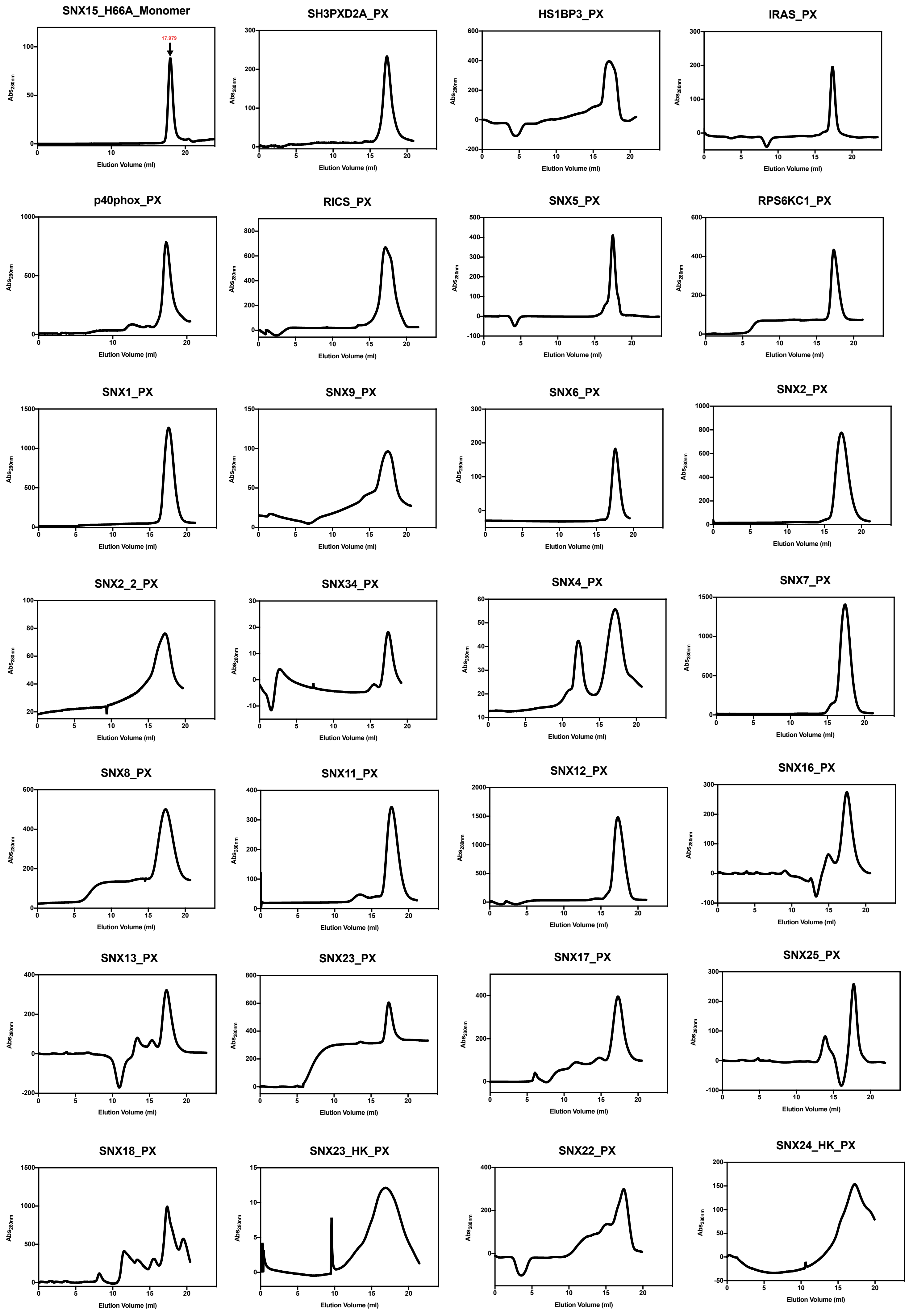
SNX15_H66A_Monomer
17.979
SH3PXD2A_PX
HS1BP3_PX
IRAS_PX
p40phox_PX
RICS_PX
SNX5_PX
RPS6KC1_PX
SNX1_PX
SNX9_PX
SNX6_PX
SNX2_PX
SNX2_2_PX
SNX34_PX
SNX4_PX
SNX7_PX
SNX8_PX
SNX11_PX
SNX12_PX
SNX16_PX
SNX13_PX
SNX23_PX
SNX17_PX
SNX25_PX
SNX18_PX
SNX23_HK_PX
SNX22_PX
SNX24_HK_PX
Abs_{280nm}
Elution Volume (ml)

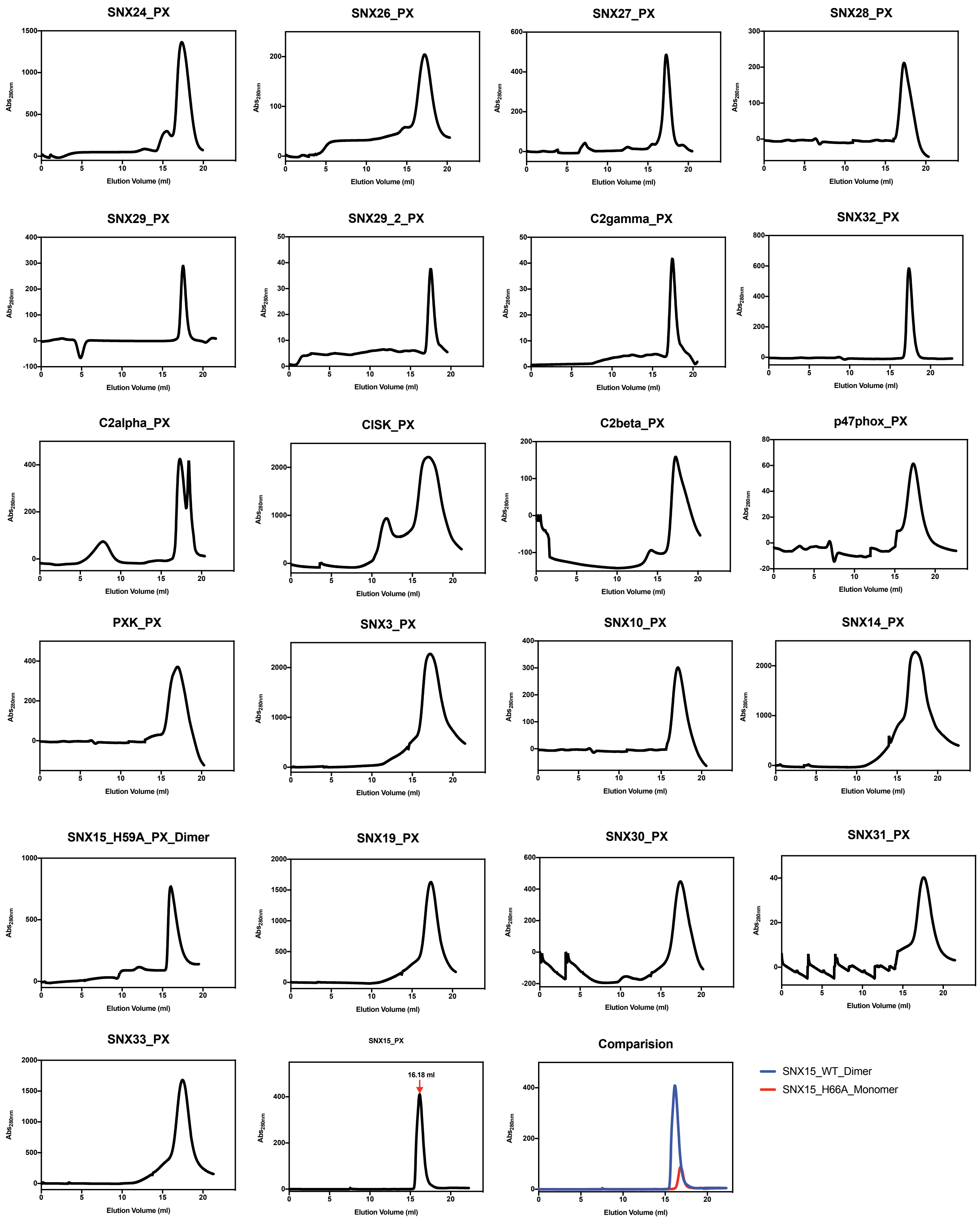

Figure S1. Gel filtration profiles of purified PX domain used in this study.
Affinity purified PX domain proteins with GST-tags removed were examined by gel filtration on a Superdex200 10/30 column.

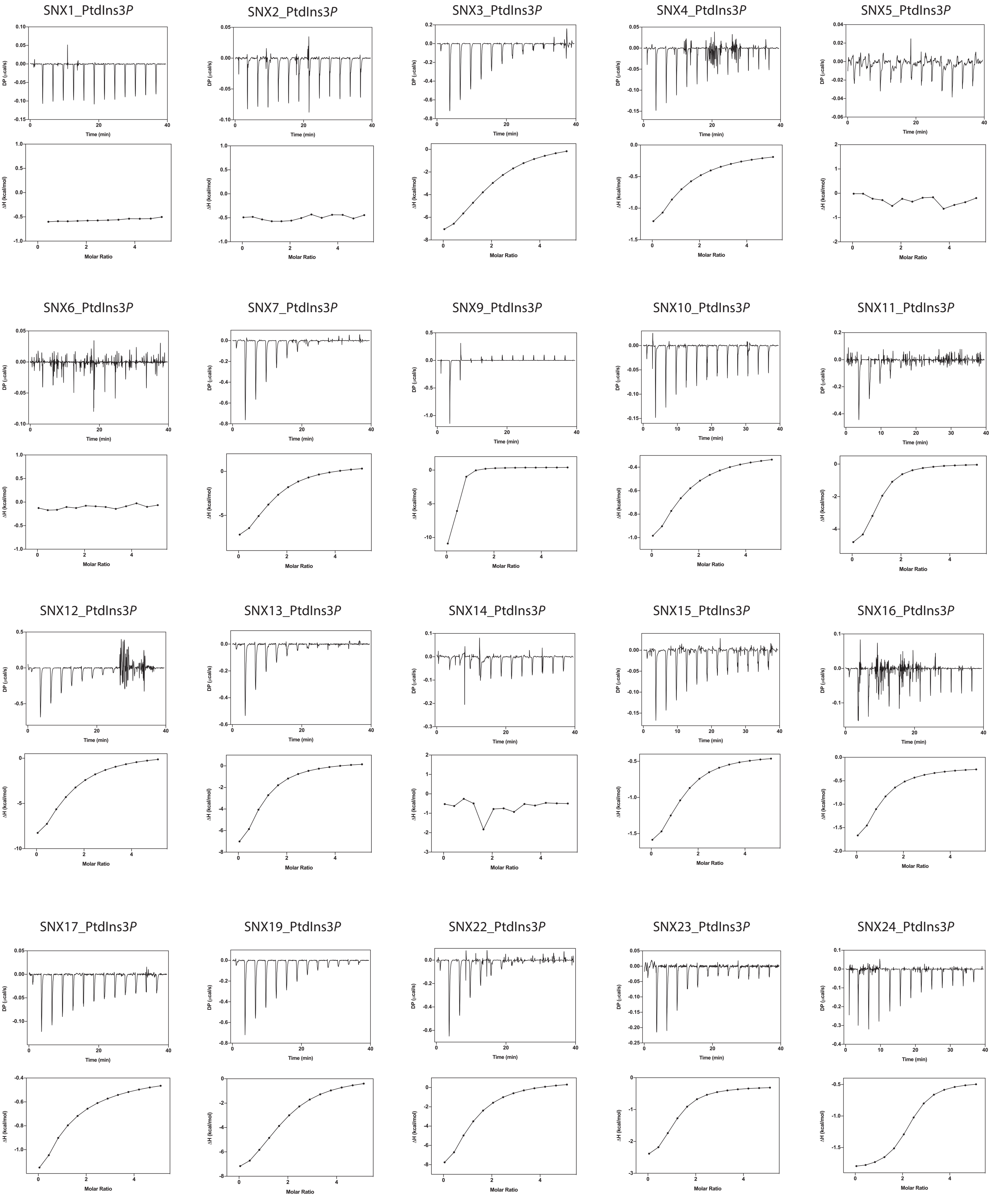
SNX1_PtdIns3P
SNX2_PtdIns3P
SNX3_PtdIns3P
SNX4_PtdIns3P
SNX5_PtdIns3P
SNX6_PtdIns3P
SNX7_PtdIns3P
SNX9_PtdIns3P
SNX10_PtdIns3P
SNX11_PtdIns3P
SNX12_PtdIns3P
SNX13_PtdIns3P
SNX14_PtdIns3P
SNX15_PtdIns3P
SNX16_PtdIns3P
SNX17_PtdIns3P
SNX19_PtdIns3P
SNX22_PtdIns3P
SNX23_PtdIns3P
SNX24_PtdIns3P
DP (μcal/s)
Time (min)
ΔH (kcal/mol)
Molar Ratio

PtdIns3*P*

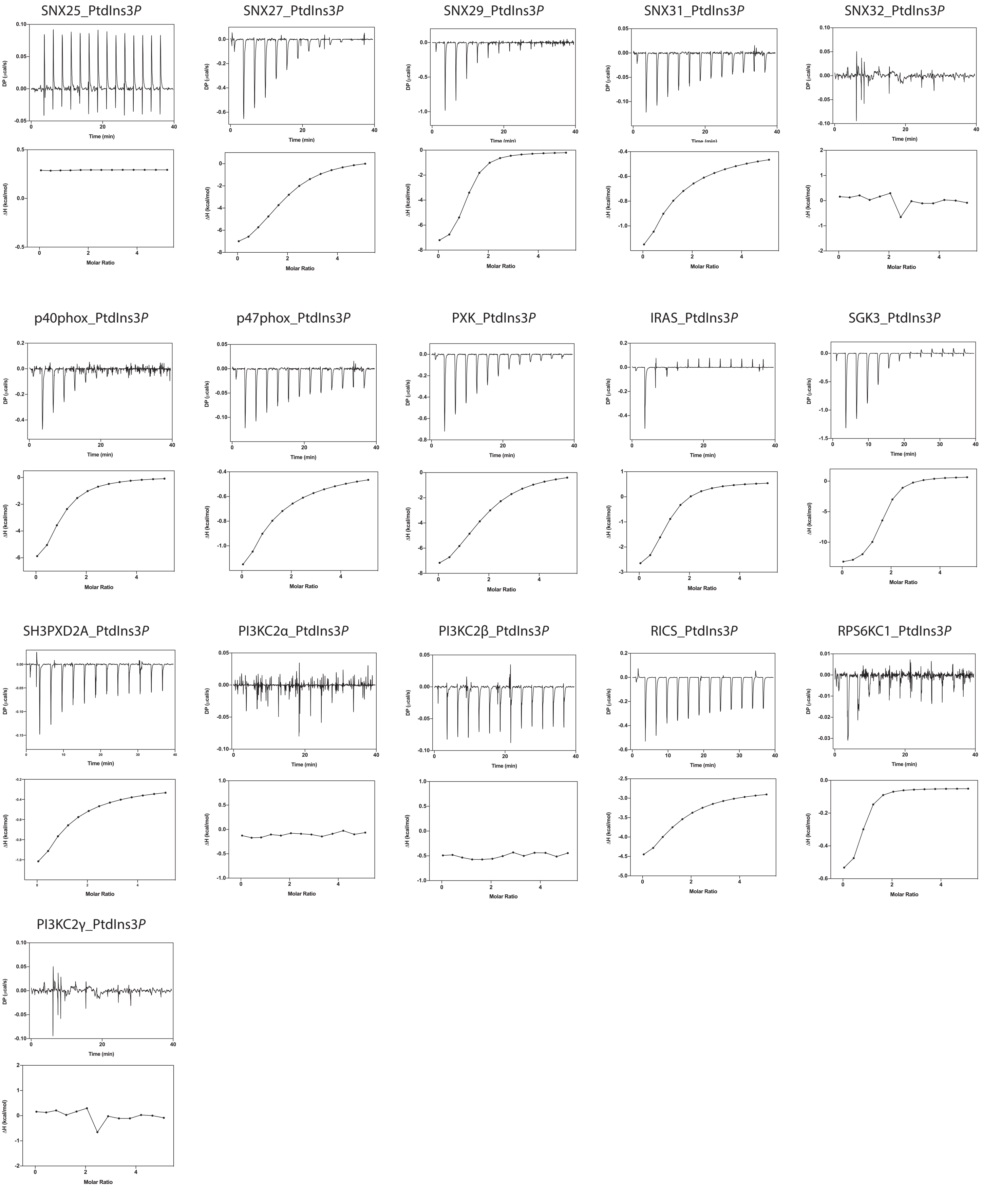
SNX25_PtdIns3P
SNX27_PtdIns3P
SNX29_PtdIns3P
SNX31_PtdIns3P
SNX32_PtdIns3P
p40phox_PtdIns3P
p47phox_PtdIns3P
PXK_PtdIns3P
IRAS_PtdIns3P
SGK3_PtdIns3P
SH3PXD2A_PtdIns3P
PI3KC2α_PtdIns3P
PI3KC2β_PtdIns3P
RICS_PtdIns3P
RPS6KC1_PtdIns3P
PI3KC2γ_PtdIns3P
DP (μcal/s)
Time (min)
ΔH (kcal/mol)
Molar Ratio

PtdIns3*P*

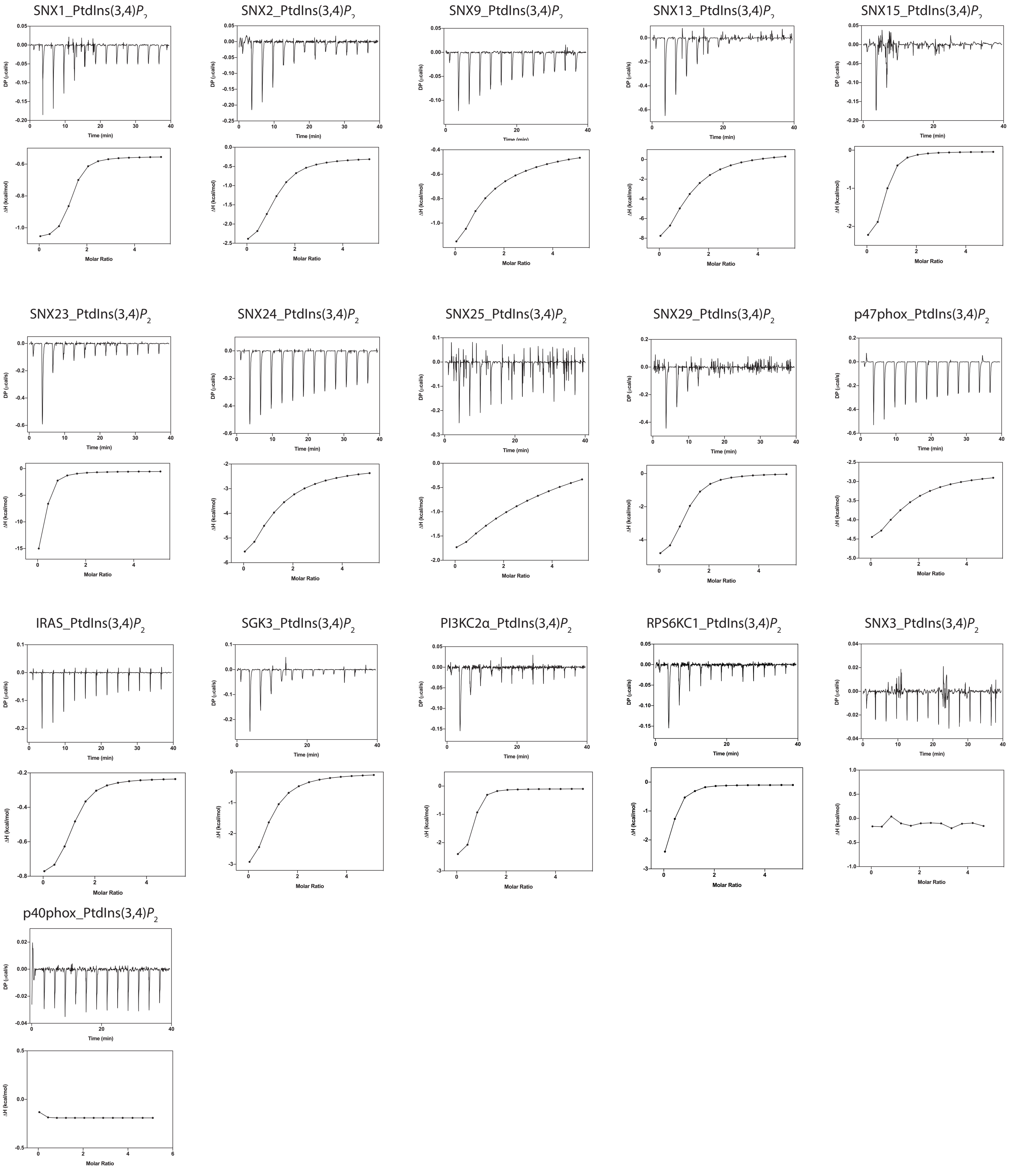
SNX1_PtdIns(3,4)P_2
SNX2_PtdIns(3,4)P_2
SNX9_PtdIns(3,4)P_2
SNX13_PtdIns(3,4)P_2
SNX15_PtdIns(3,4)P_2
SNX23_PtdIns(3,4)P_2
SNX24_PtdIns(3,4)P_2
SNX25_PtdIns(3,4)P_2
SNX29_PtdIns(3,4)P_2
p47phox_PtdIns(3,4)P_2
IRAS_PtdIns(3,4)P_2
SGK3_PtdIns(3,4)P_2
PI3KC2α_PtdIns(3,4)P_2
RPS6KC1_PtdIns(3,4)P_2
SNX3_PtdIns(3,4)P_2
p40phox_PtdIns(3,4)P_2
DP (μcal/s)
Time (min)
ΔH (kcal/mol)
Molar Ratio

PtdIns(3,4)P_2

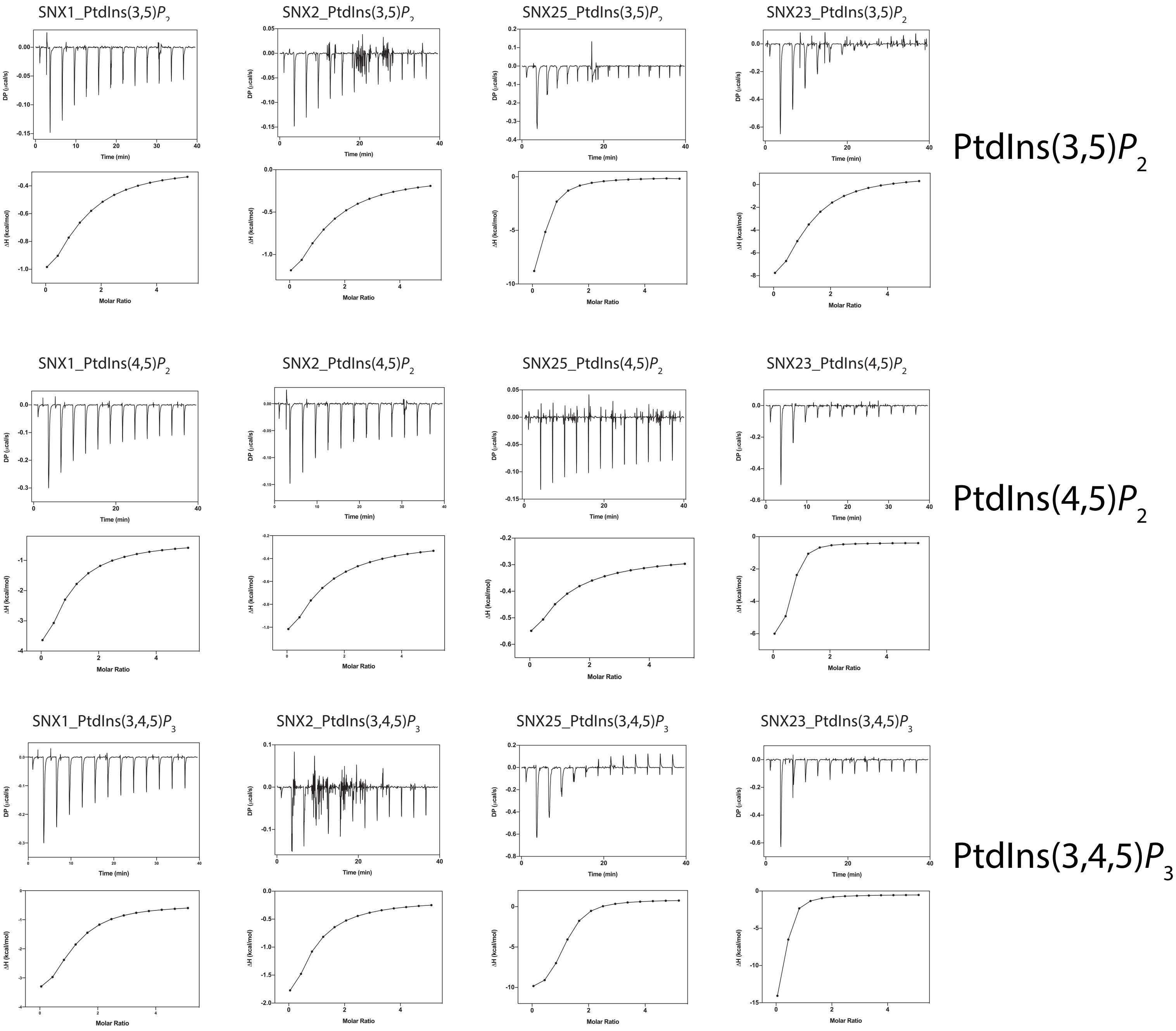

Figure S2. PX domain binding to phosphoinositides measured by ITC.
Binding of water-soluble phosphoinositide headgroup analogues (500 M) titrated into selected PX domain proteins (20 M) and measured by ITC. Top panels show the raw data and bottom panels represent the integrated and normalized data fit with a 1:1 binding model. The binding affinities (Kd) are provided in Supplementary Table 1.

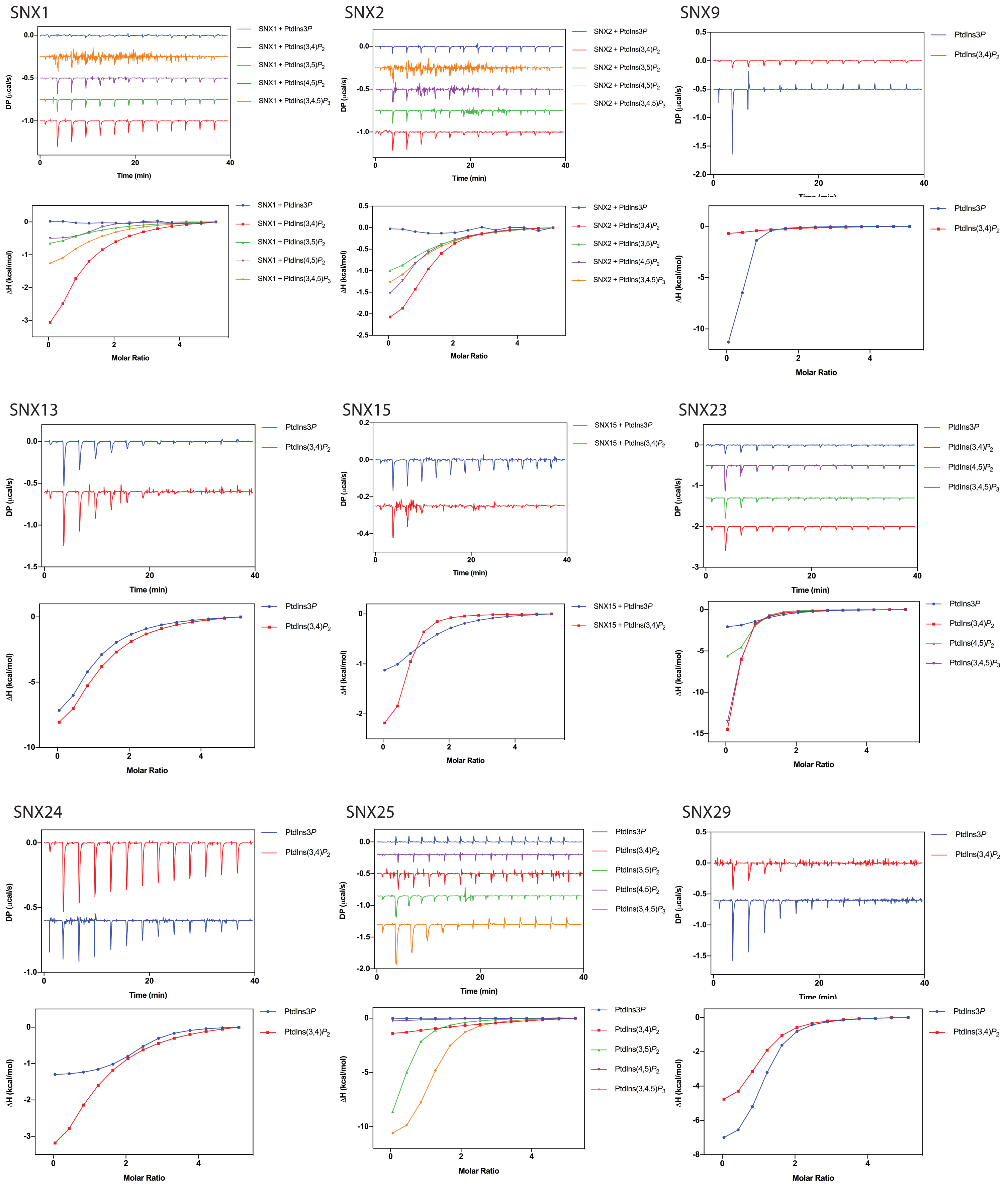

SNX1
SNX1 + PtdIns3P
SNX1 + PtdIns(3,4)P_2
SNX1 + PtdIns(3,5)P_2
SNX1 + PtdIns(4,5)P_2
SNX1 + PtdIns(3,4,5)P_3
DP (μcal/s)
Time (min)
ΔH (kcal/mol)
Molar Ratio
SNX2
SNX2 + PtdIns3P
SNX2 + PtdIns(3,4)P_2
SNX2 + PtdIns(3,5)P_2
SNX2 + PtdIns(4,5)P_2
SNX2 + PtdIns(3,4,5)P_3
SNX9
PtdIns3P
PtdIns(3,4)P_2
SNX13
PtdIns3P
PtdIns(3,4)P_2
SNX15
SNX15 + PtdIns3P
SNX15 + PtdIns(3,4)P_2
SNX23
PtdIns3P
PtdIns(3,4)P_2
PtdIns(4,5)P_2
PtdIns(3,4,5)P_3
SNX24
PtdIns3P
PtdIns(3,4)P_2
SNX25
PtdIns3P
PtdIns(3,4)P_2
PtdIns(3,5)P_2
PtdIns(4,5)P_2
PtdIns(3,4,5)P_3
SNX29
PtdIns3P
PtdIns(3,4)P_2

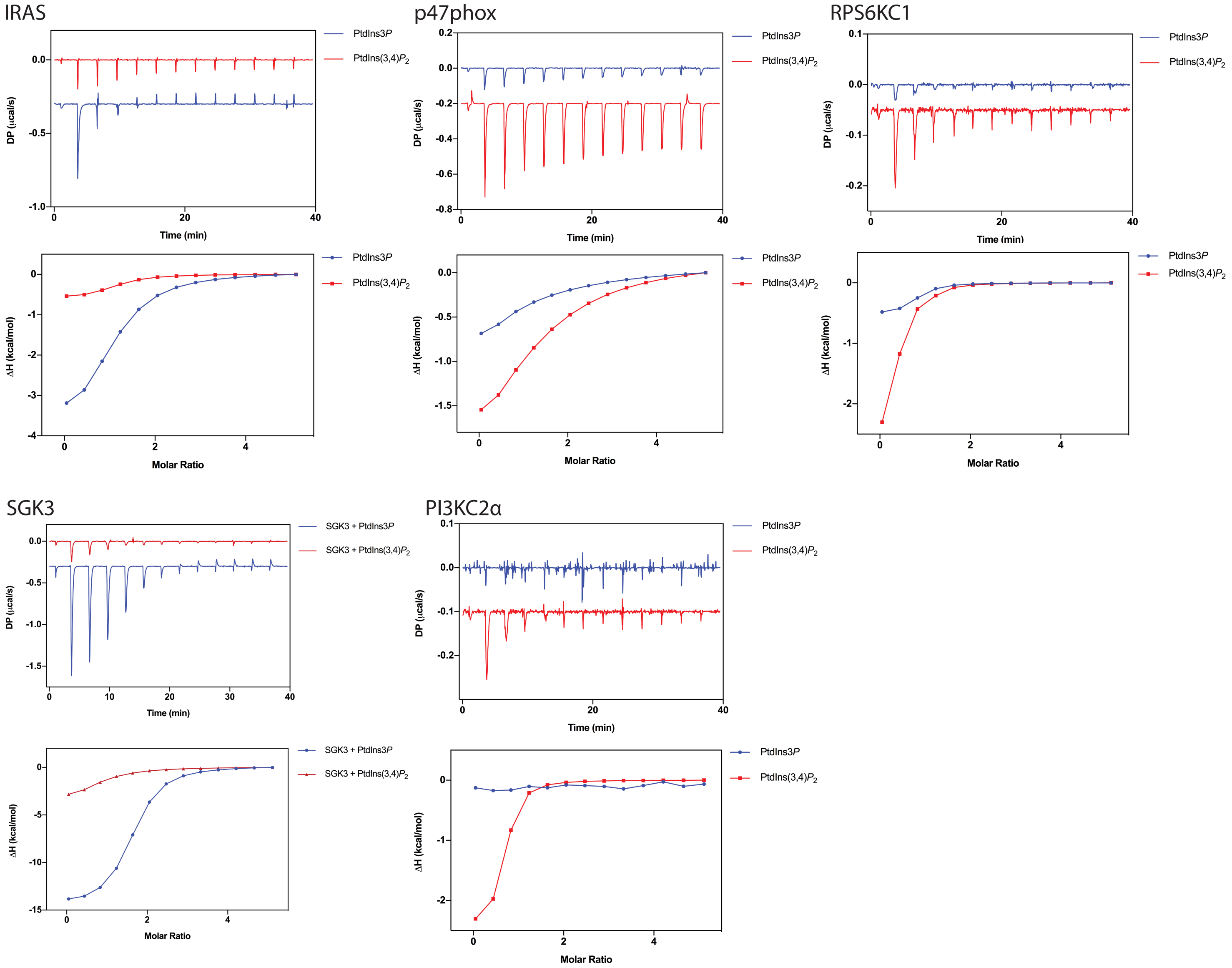

Figure S3. Comparison of PX domain binding to different phosphoinositides measured by ITC.
Binding of water-soluble phosphoinositide headgroup analogues (500 M) titrated into selected PX domain proteins (20 M) and measured by ITC. Top panels show the raw data and bottom panels represent the integrated and normalized data fit with a 1:1 binding model. The binding affinities (Kd) are provided in Supplementary Table 1.

PtdIns3*P*

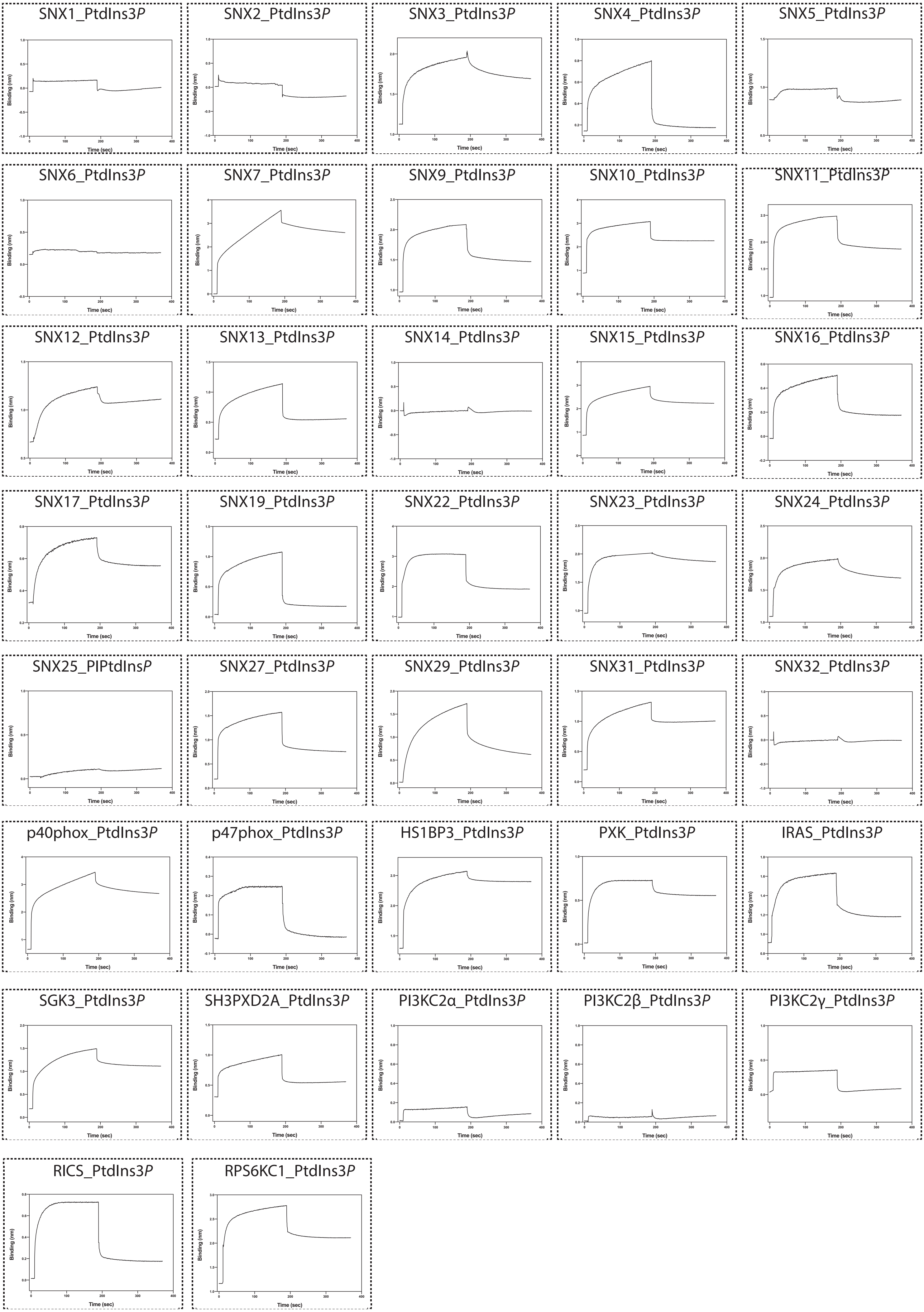
SNX1_PtdIns3P
SNX2_PtdIns3P
SNX3_PtdIns3P
SNX4_PtdIns3P
SNX5_PtdIns3P
SNX6_PtdIns3P
SNX7_PtdIns3P
SNX9_PtdIns3P
SNX10_PtdIns3P
SNX11_PtdIns3P
SNX12_PtdIns3P
SNX13_PtdIns3P
SNX14_PtdIns3P
SNX15_PtdIns3P
SNX16_PtdIns3P
SNX17_PtdIns3P
SNX19_PtdIns3P
SNX22_PtdIns3P
SNX23_PtdIns3P
SNX24_PtdIns3P
SNX25_PIPtdInsP
SNX27_PtdIns3P
SNX29_PtdIns3P
SNX31_PtdIns3P
SNX32_PtdIns3P
p40phox_PtdIns3P
p47phox_PtdIns3P
HS1BP3_PtdIns3P
PXK_PtdIns3P
IRAS_PtdIns3P
SGK3_PtdIns3P
SH3PXD2A_PtdIns3P
PI3KC2α_PtdIns3P
PI3KC2β_PtdIns3P
PI3KC2γ_PtdIns3P
RICS_PtdIns3P
RPS6KC1_PtdIns3P
Binding (nm)
Time (sec)

PtdIns(3,4)P_2

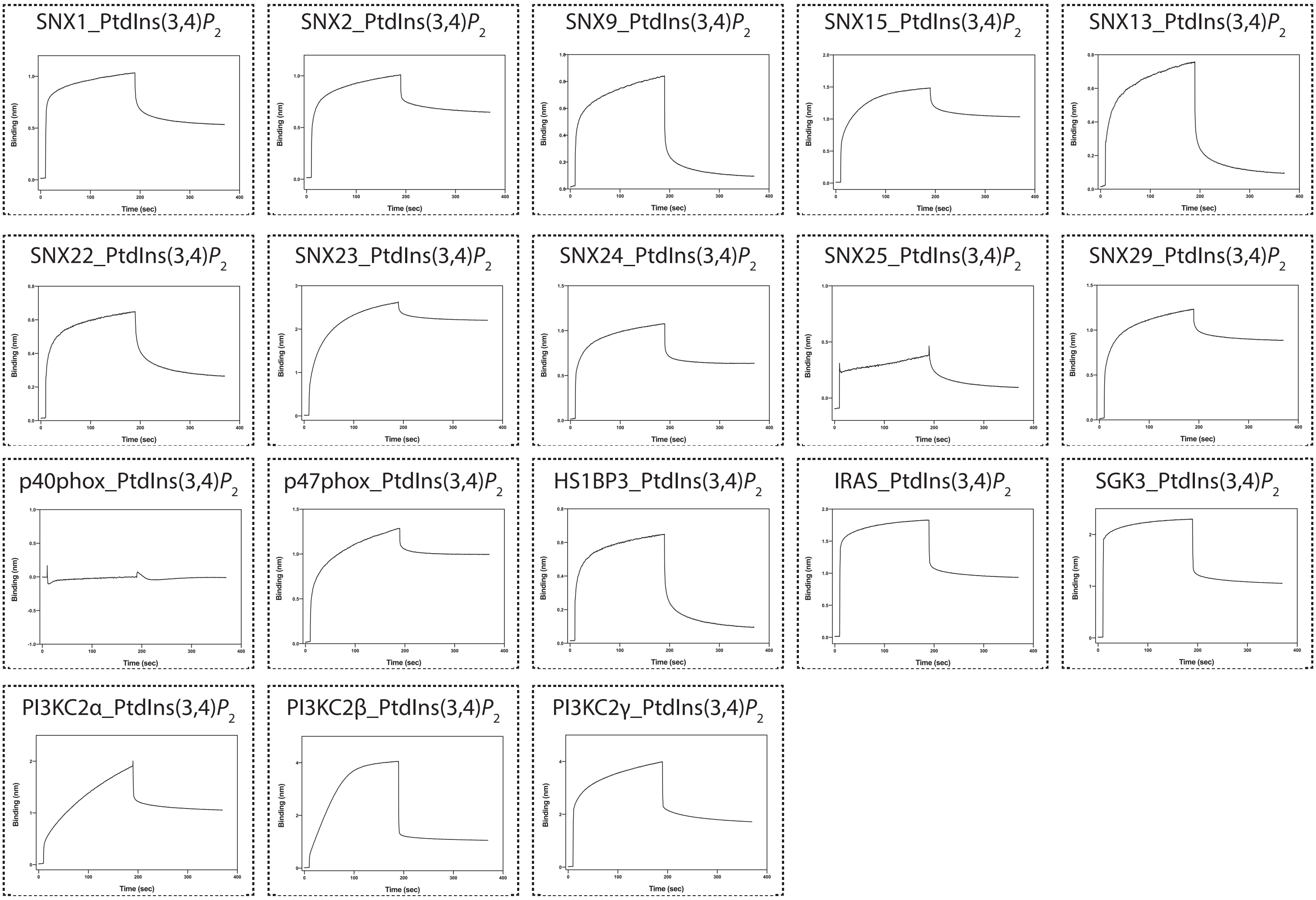

SNX1_PtdIns(3,4)P2
SNX2_PtdIns(3,4)P2
SNX9_PtdIns(3,4)P2
SNX15_PtdIns(3,4)P2
SNX13_PtdIns(3,4)P2
SNX22_PtdIns(3,4)P2
SNX23_PtdIns(3,4)P2
SNX24_PtdIns(3,4)P2
SNX25_PtdIns(3,4)P2
SNX29_PtdIns(3,4)P2
p40phox_PtdIns(3,4)P2
p47phox_PtdIns(3,4)P2
HS1BP3_PtdIns(3,4)P2
IRAS_PtdIns(3,4)P2
SGK3_PtdIns(3,4)P2
PI3KC2α_PtdIns(3,4)P2
PI3KC2β_PtdIns(3,4)P2
PI3KC2γ_PtdIns(3,4)P2
Binding (nm)
Time (sec)

PtdIns(4,5)P_2

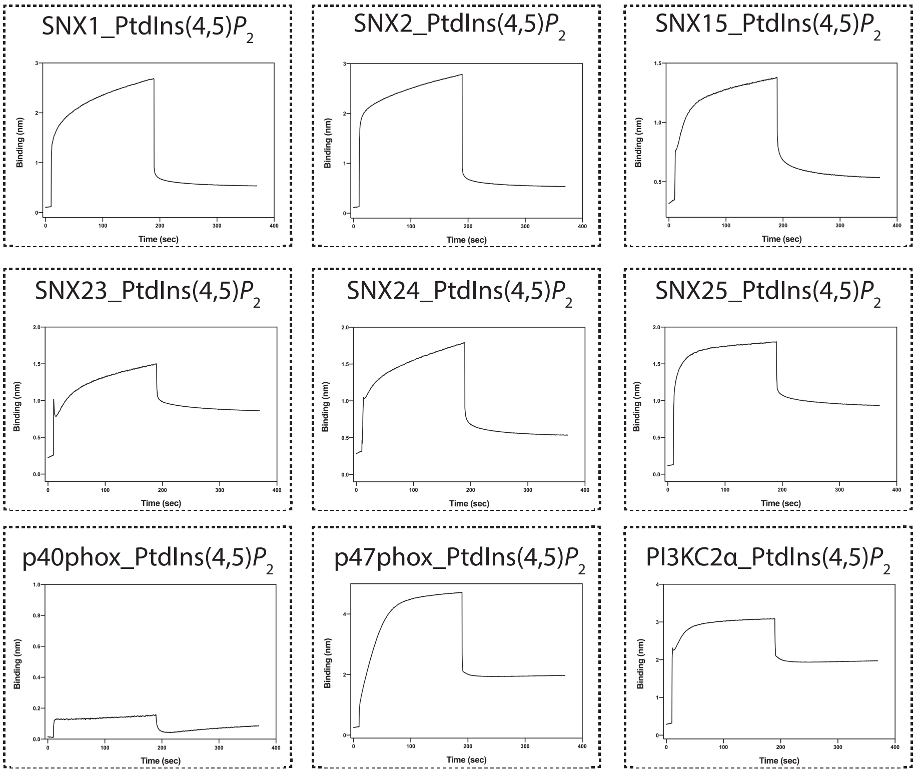

PtdIns(3,4,5)P_3

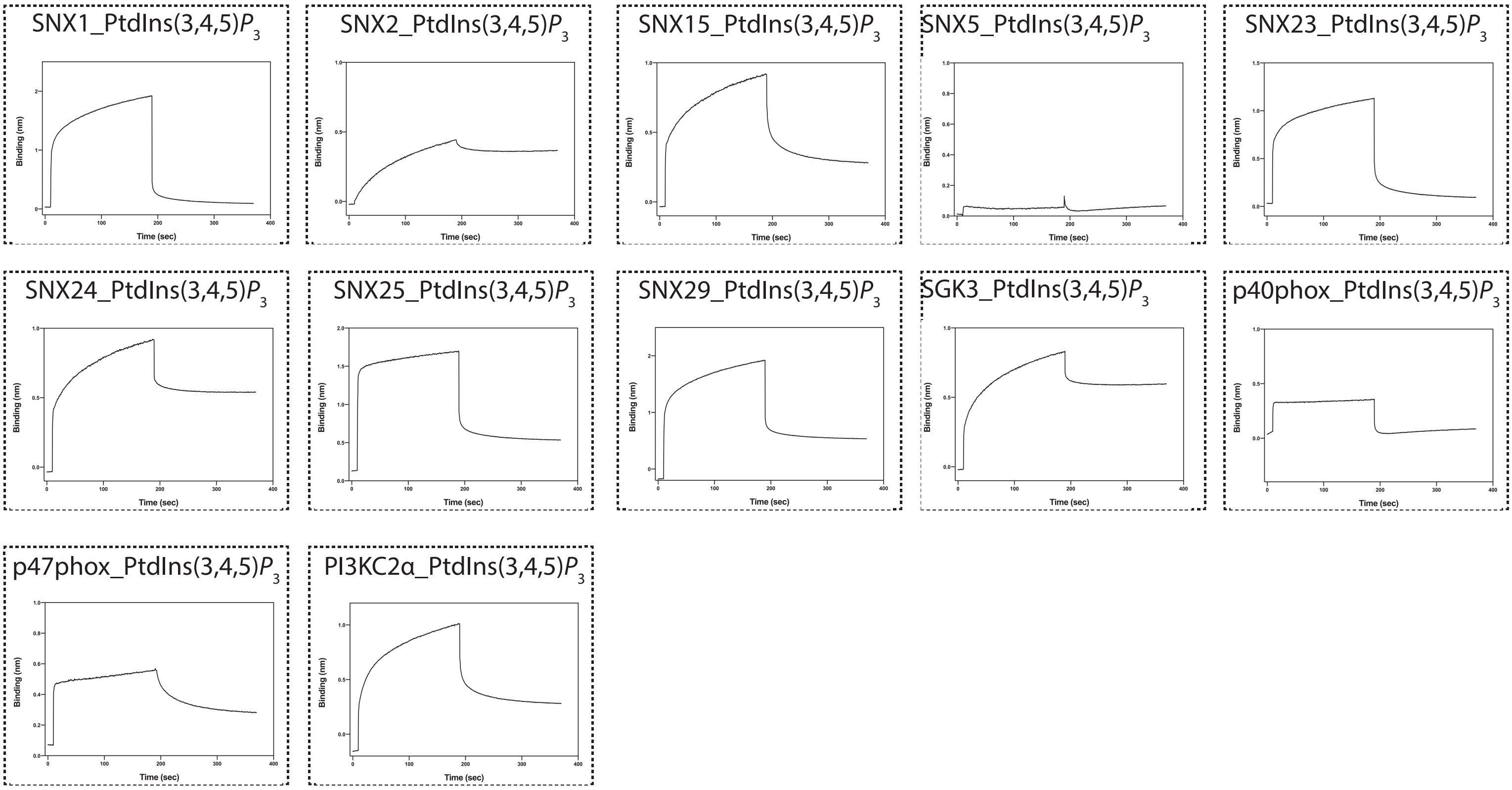

Figure S4. PX domain binding to phosphoinositides measured by BLItz.
Biotinylated PC/PE liposomes containing the indicated phosphoinositides were coupled to a streptavidin probe and binding to PX proteins measured at a single concentration of 20 M using the BLItz system. Binding kinetics were calculated using Prism software and are provided in Supplementary Table 2.

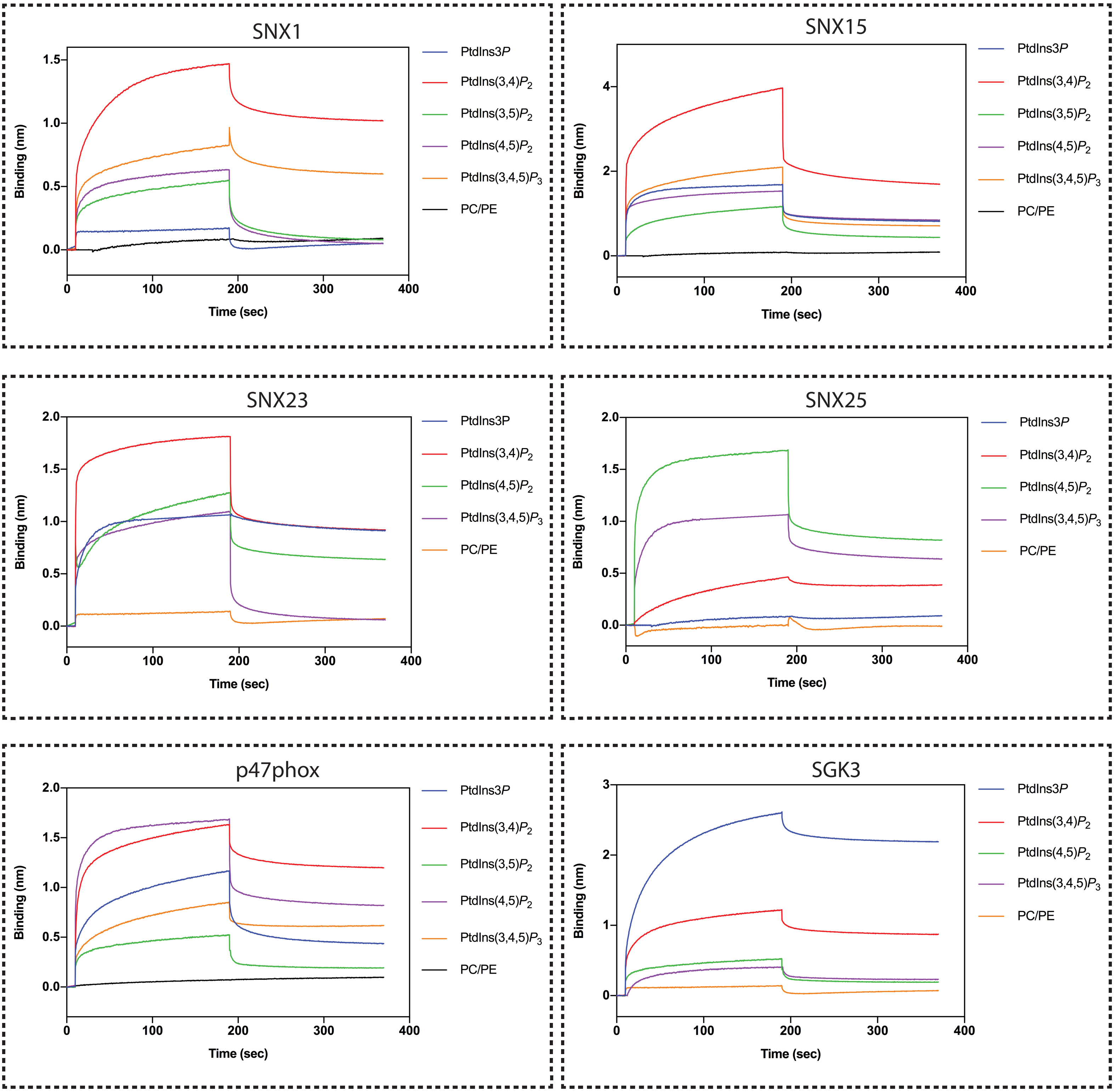

Figure S5. Comparison of PX domain binding to different phosphoinositides measured by BLItz.
Biotinylated PC/PE liposomes containing the indicated phosphoinositides were coupled to a streptavidin probe and binding to PX proteins measured at a single concentration of 20 M using the BLItz system. Binding kinetics were calculated using Prism software and are provided in Supplementary Table 2.

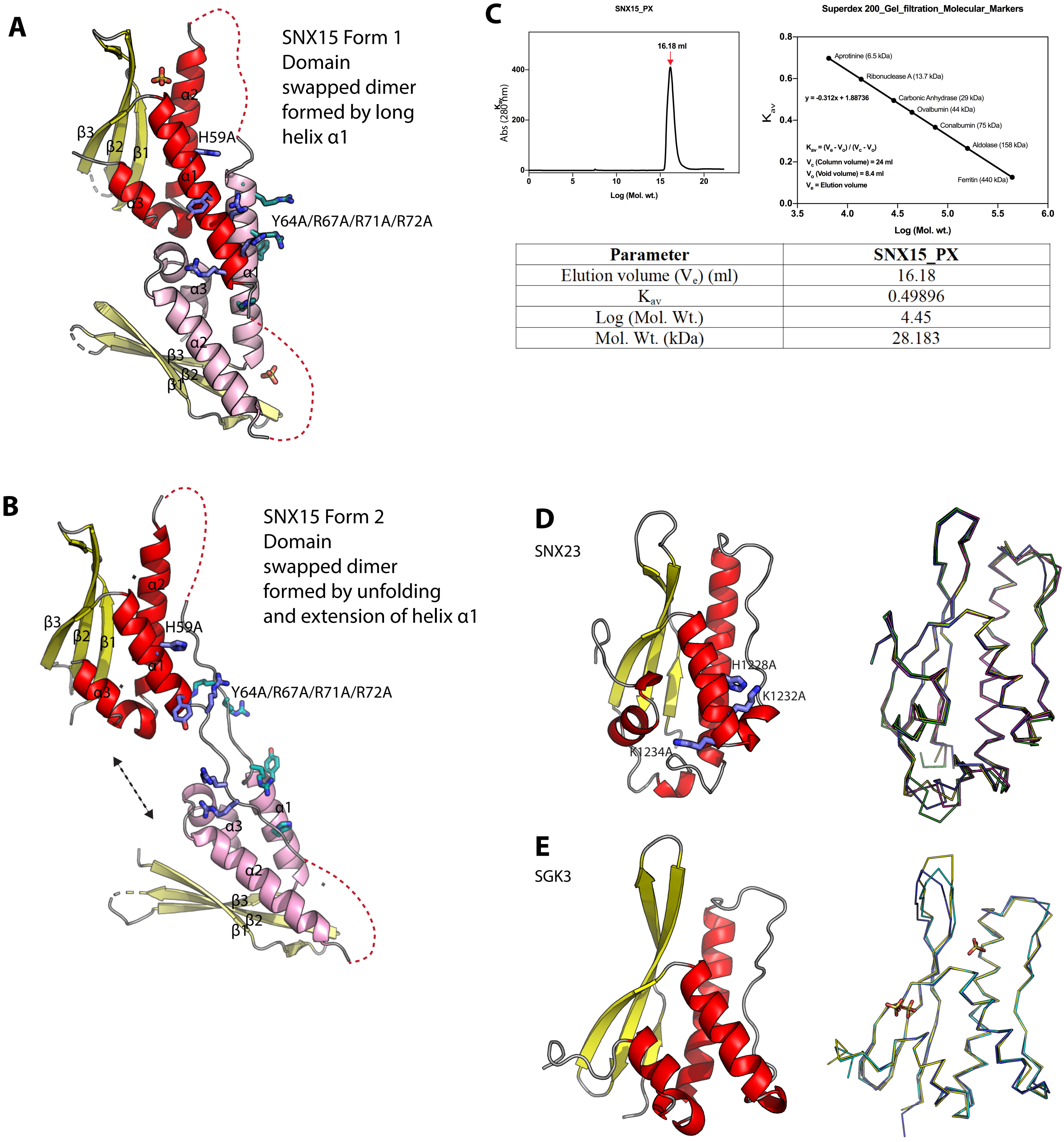

Parameter	SNX15_PX
Elution volume (V_e) (ml)	16.18
K_{av}	0.49896
Log (Mol. Wt.)	4.45
Mol. Wt. (kDa)	28.183

Figure S6. Crystal structures of the SNX15, SNX23, SGK3, and SNX32 PX domains.

(A) Cartoon representation of the PX domain of SNX15 bound to sulphate (crystal form 1). The protein forms a domain-swapped dimer, whereby the 1 helix is longer than normal leading to swapping of the subsequent 2 and 3 with an adjacent molecule in the crystal lattice. (B) Cartoon representation of the PX domain of SNX15 (crystal form 2). The protein still forms a domain-swapped dimer, but the C-terminus of the 1 helix unfolds to form a longer extended structure. (C) Analytical gel filtration profile of the SNX15 PX domain (compared with the standard calibration curve). The solid line represents the normalized UV absorbance at 280 nm. The chromatogram peak represents the corresponding measured molecular weight in kDa. (D) (Left) Cartoon representation of the SNX23/Kif16B PX domain. (Right) Overlay of the three SNX23 chains within the asymmetric unit with the previous SNX23 PX domain structure in complex with sulphate (PDB ID 2V14). (E) (Left) Cartoon representation of the SGK3 PX domain. (Right) Overlay of the SGK3 structure with the two chains of the asymmetric unit within the previous SGK3 PX domain structure (PDB ID 1XTN).

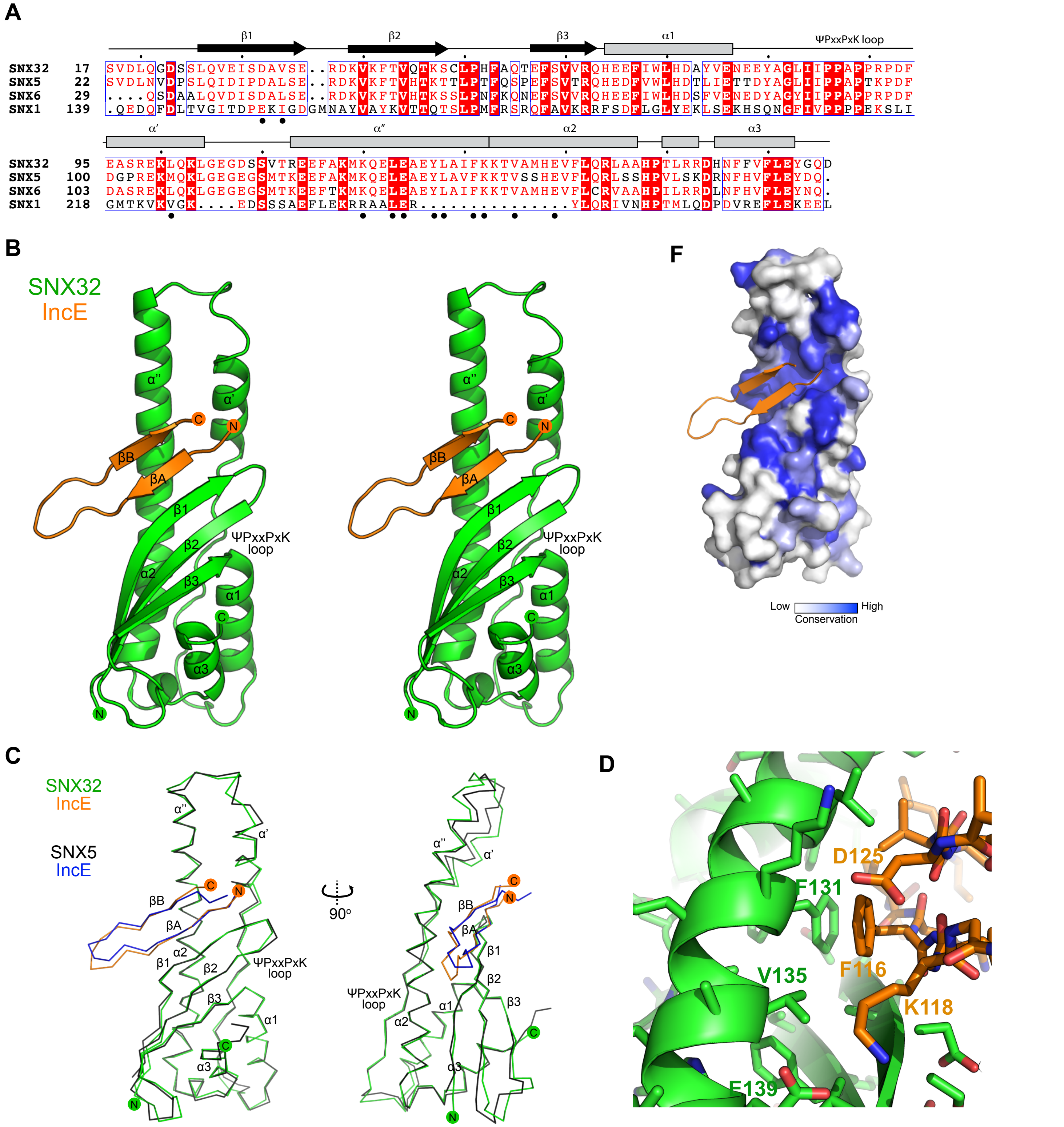

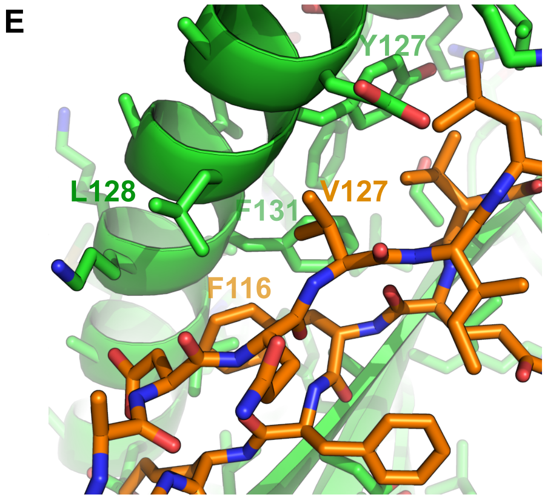

Figure S7. Sequence and crystal structure of the SNX32 PX domain in complex with IncE.

(A) Sequence alignment of human SNX32, SNX5, SNX6 and SNX1 PX domains. Conserved residues are indicated in red. Side-chains that directly interact with IncE in the crystal structure are indicated with black circles. Alignment was made with ESPRIPT [20]. (B) Cartoon diagram (shown in wall-eye stereo) showing the crystal structure of the human SNX32 PX domain (green) in complex with IncE (residues 108-132) (orange). All structure images were generated using PyMOL (Delano Scientific). The extended helix-turn-helix structure composed of helices ′ and ″ extends towards to the top of the image. (C) Superposition of the crystal structure of the SNX32 PX domain complex with IncE (green and orange) with that of the previous SNX5-IncE structure (black and blue) (PDB ID 5TGI) [10]. Structures are shown in backbone ribbon representation. (D and E) Two close up views of the SNX32-IncE interface highlighting the important interactions between the two proteins. (F) The relative sequence conservation of SNX32 side-chains are plotted on the surface representation from blue (highly conserved) to white (not conserved). The IncE peptide is shown in orange cartoon representation. The conservation was calculated using CONSURF [21].

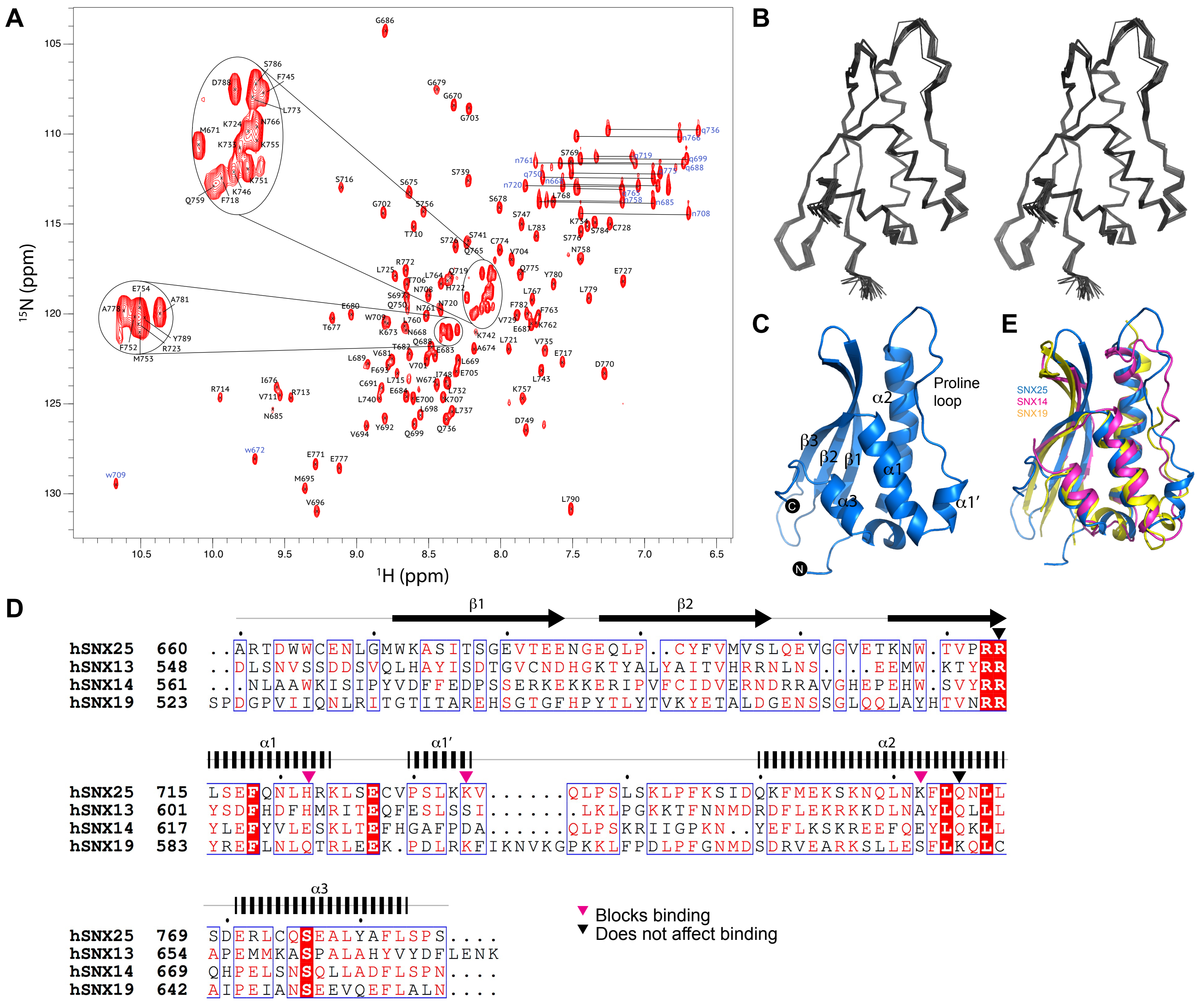

Figure S8. NMR structure of the SNX25 PX domain.
(A) 2D 1H-15N-HSQC spectra of the SNX25 PX domain at 500 M with sequence-specific assignments shown. (B) The 20 lowest energy NMR structures calculated for the SNX25 PX domain, shown in wall-eye stereo. (C) Solution NMR structure of the SNX25 PX domain in ribbon diagram. (D) Sequence alignment of the SNX25 PX domain with the other human RGS-PX family members. Secondary structure is indicated based on the SNX25 NMR structure. (E) Structural overlay of the SNX25 PX domain NMR structure with the SNX14 and SNX19 PX domains determined by X-ray crystallography (PDB IDs 4P2J and 4PQO respectively) (Mas et al., 2014).

		Canonical				Non-Canonical		
		R	Y	K	R	H/Y	K/R	Group
PX-BAR	SNX1	R	F	K	R	Y	KK	III
	SNX2	R	F	K	R	H	KK	III
	SNX4	R	Y	K	R	R		I
	SNX5	Q	H	K	T	H		I
	SNX6	Q	H	R	T	H		I
	SNX7	R	Y	K	R	K	K	II
	SNX8	R	Y	K	R	Q	KR	ii
	SNX30	R	Y	K	R	R	K	ii
	SNX32	Q	H	R	T	H		I
SH3-PX-BAR	SNX9	R	Y	K	R	Y	RK	IV
	SNX18	R	Y	K	R	Y	RK	iv
	SNX33	R	Y	K	R	Y	RK	iv
RGS-PX	SNX13	R	Y	K	R	H	R	IV
	SNX14	R	Y	K	K	E	K	I
	SNX19	R	Y	K	R	Q	RKRKK	II
	SNX25	R	L	K	K	H	RKKK	III
PX-FERM	SNX17	R	Y	K	R	H	RK	II
	SNX27	R	Y	K	R	H	RK	II
	SNX31	R	Y	K	R	N	RR	II
PX-only	SNX3	R	Y	K	K	R	RK	II
	SNX10	R	Y	K	R	R	R	II
	SNX11	R	Y	K	R	R	KR	IV
	SNX12	R	Y	K	R	K	RK	II
	SNX22	R	Y	K	R	H	KRKKK	IV
	SNX24	R	Y	K	R	H	KKK	IV
	HS1BP3	K	Y	K	K	Y	KR	IV
PX-SH3	SH3PXD2A	R	Y	K	R	Q	KKKR	II
	SH3PXD2B	R	Y	K	R	Q	KKKR	ii
	SNX28	S	W	L	R	Q	KKRR	I
	p40phox	R	Y	K	R	Q	KRK	II
	p47phox	R	F	P	R	H	KKR	IV
PX-S/T kinase	PXK	R	Y	K	R	N		II
	RPS6KC1	R	Y	K	R	H	KKR	IV
	SGK3	R	Y	K	R	Y	KK	IV
PX-SH3-GAP	SNX26	S	Y	P	V	D	RRR	I
	PX-RICS	S	Y	R	V	D	KRR	IV
PX-PI3-kinase	PIK3C2A	T	F	R	R	H	KK	III
	PIK3C2B	T	F	R	R	H	KR	III
	PIK3C2G	S	F	H	R	H	K	III
PX-PH-PLD	PLD1	K	F	R	R	H	RKKRRRRR	iii
	PLD2	K	Y	R	K	H	RRKRRR	iii
PX-PXB	SNX20	R	Y	K	R	Q	KKR	iv
	SNX21	R	Y	K	R	H	RRR	iv
Kinesin-PX	SNX23	R	Y	K	R	H	KKK	IV
PX-MIT	SNX15	R	Y	R	R	H	RRR	IV
PX-LRR-IRAS	IRAS	R	Y	K	R	H	KRKK	IV
PX-SNX16	SNX16	R	Y	K	Q	N	KK	II
SNX29-PX	SNX29	R	Y	K	R	H	KKR	IV
PX-SNX34	SNX34	R	S	R	R	W	RR	i

Figure S9. Conservation of canonical and secondary site amino-acid sequences needed for phosphoinositide binding. Black and grey indicates non-conservative and conservative substitutions respectively. The phosphoinositide-binding group is indicated in the last column, and lower case grey letters indicate predictions of the phosphoinositide-binding preference based on the sequences of the proteins.

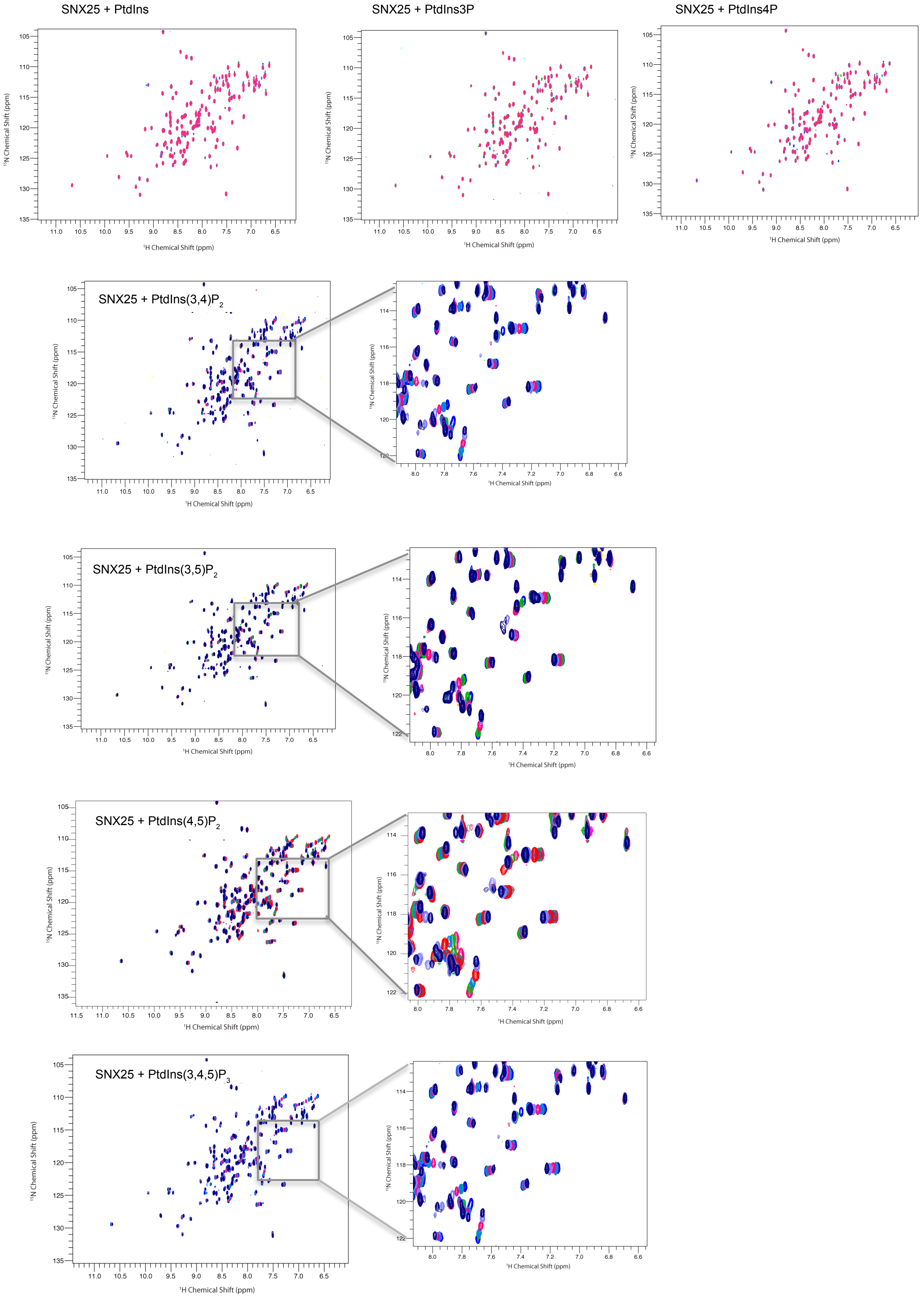

Figure S10. Binding of SNX25 to phosphoinositides by NMR.
2D 1H-15N-HSQC spectra of the SNX25 PX domain at 50   M were recorded in the presence of increasing molar ratios of the indicated di-C8 soluble phosphoinositide species. PtdIns and mono-phosphorylated PtdIns3P and PtdIns4P did not show significant binding. Di and tri-phosphorylated species however resulted in significant chemical shift perturbations.

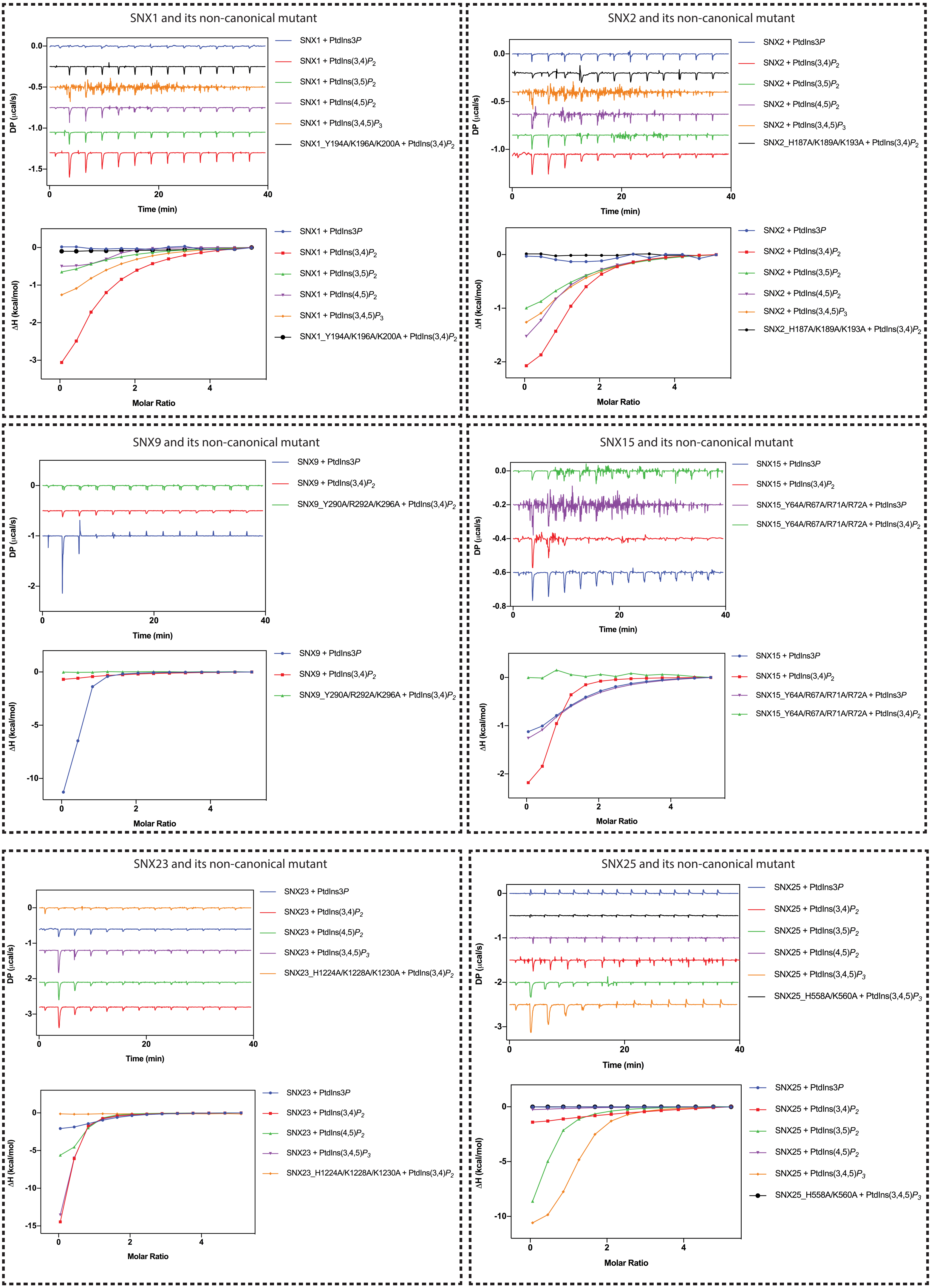

SNX1 and its non-canonical mutant
SNX1 + PtdIns3P
SNX1 + PtdIns(3,4)P2
SNX1 + PtdIns(3,5)P2
SNX1 + PtdIns(4,5)P2
SNX1 + PtdIns(3,4,5)P3
SNX1_Y194A/K196A/K200A + PtdIns(3,4)P2
DP (μcal/s)
Time (min)
ΔH (kcal/mol)
Molar Ratio
SNX2 and its non-canonical mutant
SNX2 + PtdIns3P
SNX2 + PtdIns(3,4)P2
SNX2 + PtdIns(3,5)P2
SNX2 + PtdIns(4,5)P2
SNX2 + PtdIns(3,4,5)P3
SNX2_H187A/K189A/K193A + PtdIns(3,4)P2
SNX9 and its non-canonical mutant
SNX9 + PtdIns3P
SNX9 + PtdIns(3,4)P2
SNX9_Y290A/R292A/K296A + PtdIns(3,4)P2
SNX15 and its non-canonical mutant
SNX15 + PtdIns3P
SNX15 + PtdIns(3,4)P2
SNX15_Y64A/R67A/R71A/R72A + PtdIns3P
SNX15_Y64A/R67A/R71A/R72A + PtdIns(3,4)P2
SNX23 and its non-canonical mutant
SNX23 + PtdIns3P
SNX23 + PtdIns(3,4)P2
SNX23 + PtdIns(4,5)P2
SNX23 + PtdIns(3,4,5)P3
SNX23_H1224A/K1228A/K1230A + PtdIns(3,4)P2
SNX25 and its non-canonical mutant
SNX25 + PtdIns3P
SNX25 + PtdIns(3,4)P2
SNX25 + PtdIns(3,5)P2
SNX25 + PtdIns(4,5)P2
SNX25 + PtdIns(3,4,5)P3
SNX25_H558A/K560A + PtdIns(3,4,5)P3

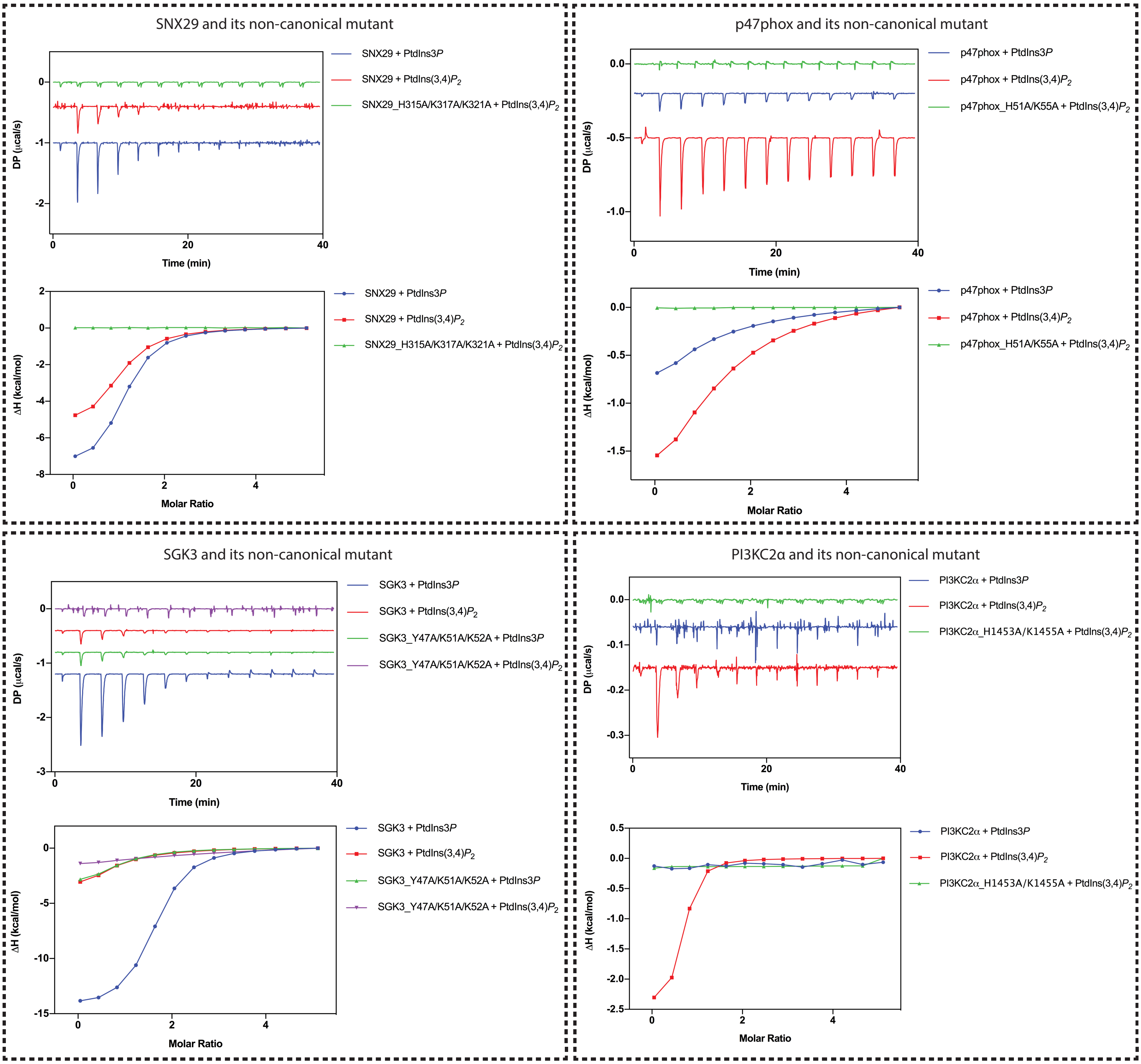

Figure S11. PX domain mutants binding to phosphoinositides measured by ITC.
Binding of water-soluble phosphoinositide headgroup analogues (500 M) titrated into selected PX domain proteins and their mutants (20 M) measured by ITC. Top panels show the raw data and bottom panels represent the integrated and normalized data fit with a 1:1 binding model. The binding affinities (Kd) are provided in Supplementary Table 1.

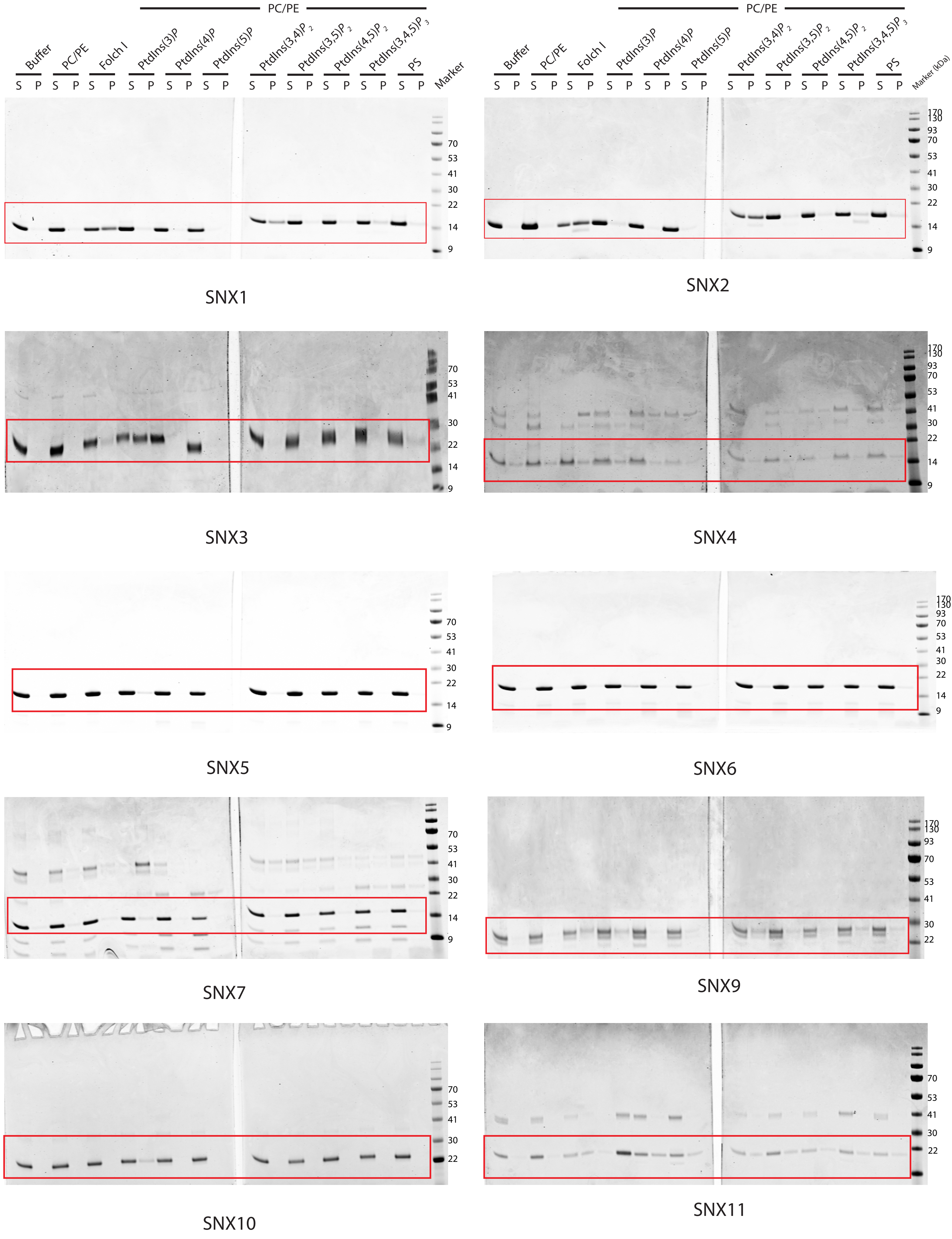

Raw gels related to Figure 1

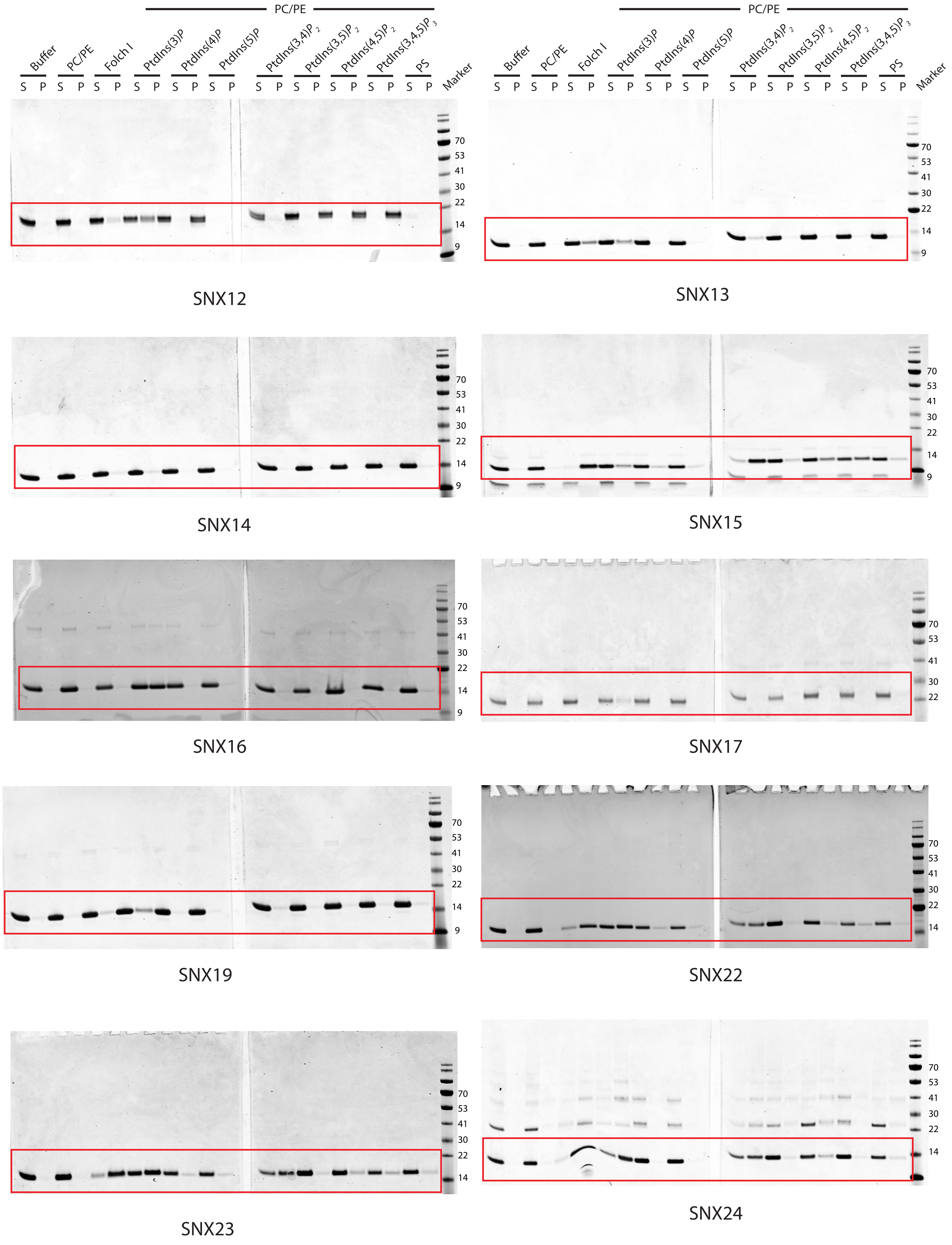

Raw gels related to Figure 1

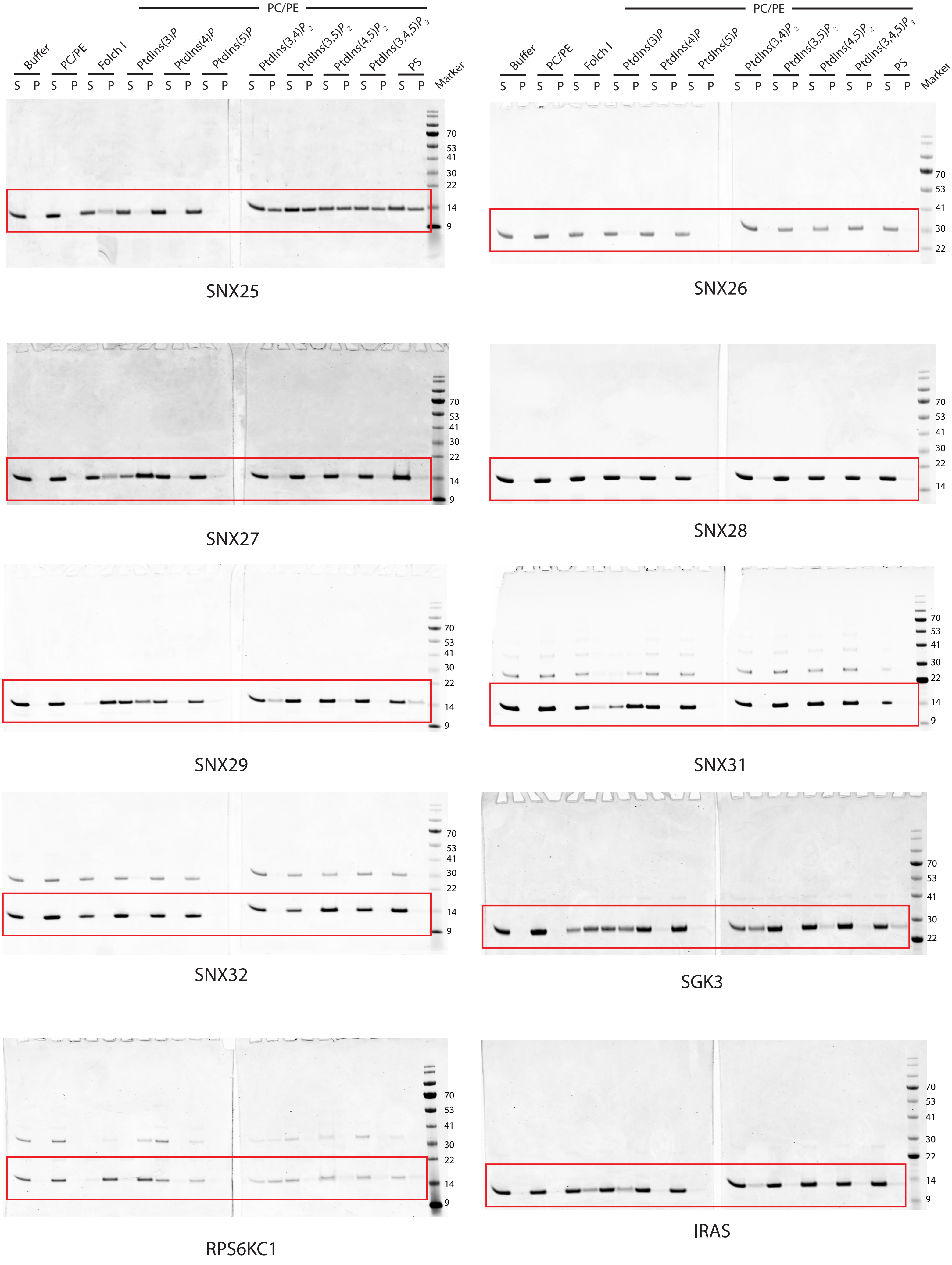

Raw gels related to Figure 1

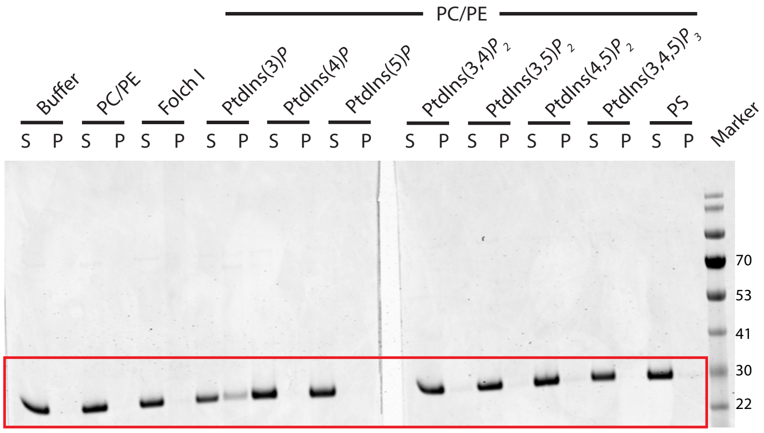

p40phox

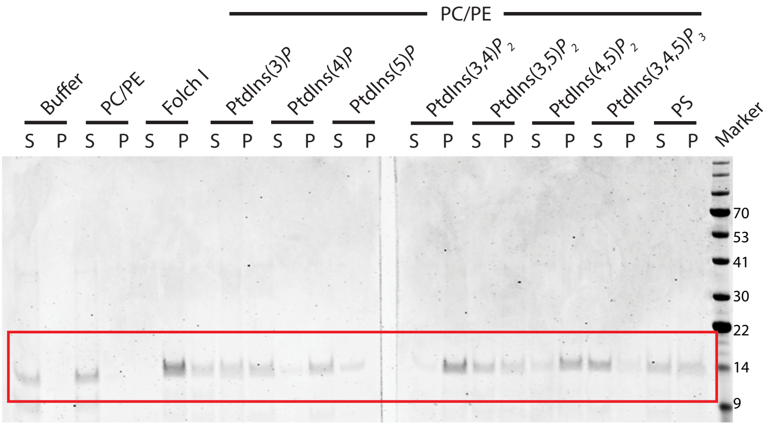

p47phox

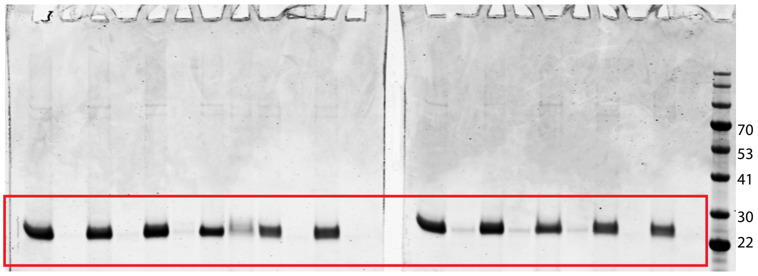

PXK

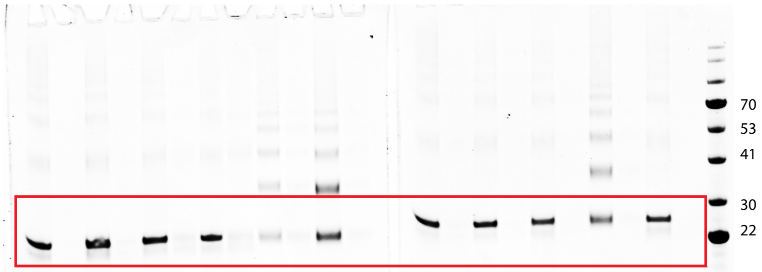

RICS

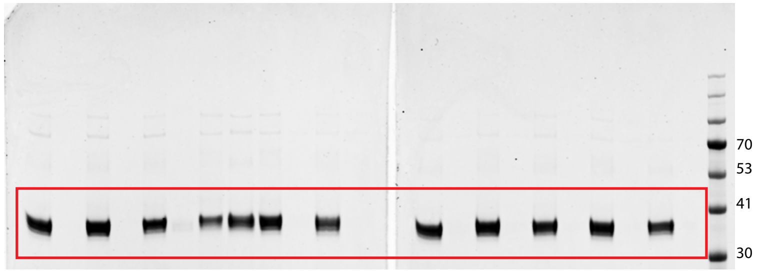

SH3PXD2A

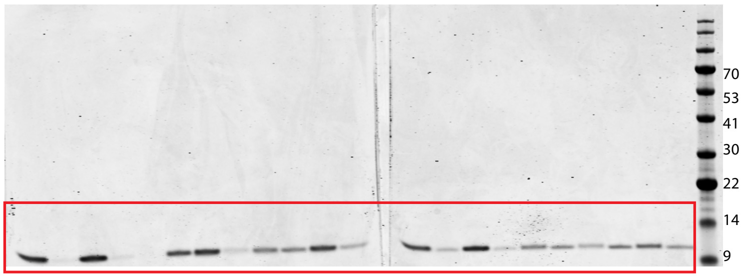

HS1BP3

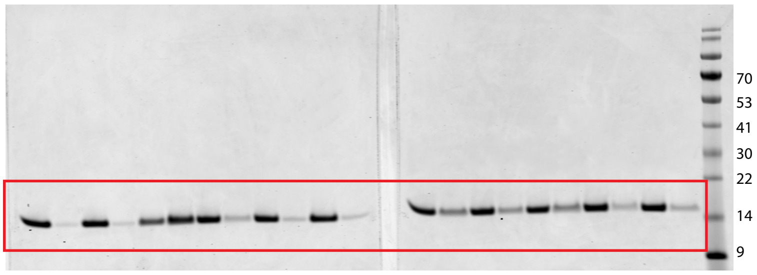

PI3KC2α

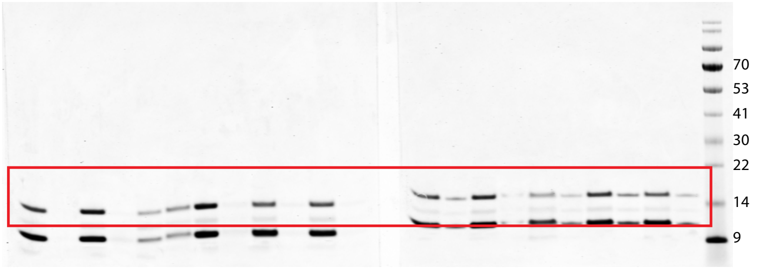

PI3KC2β

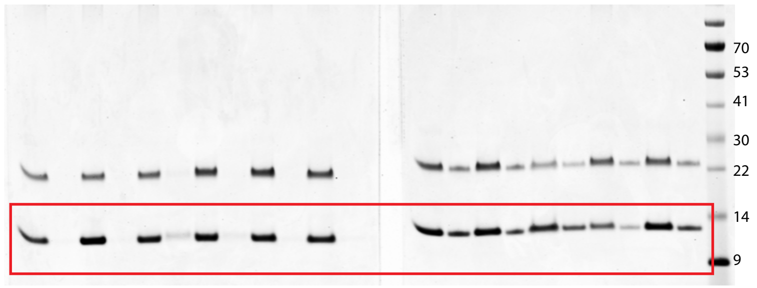

PI3KC2γ

Raw gels related to Figure 1

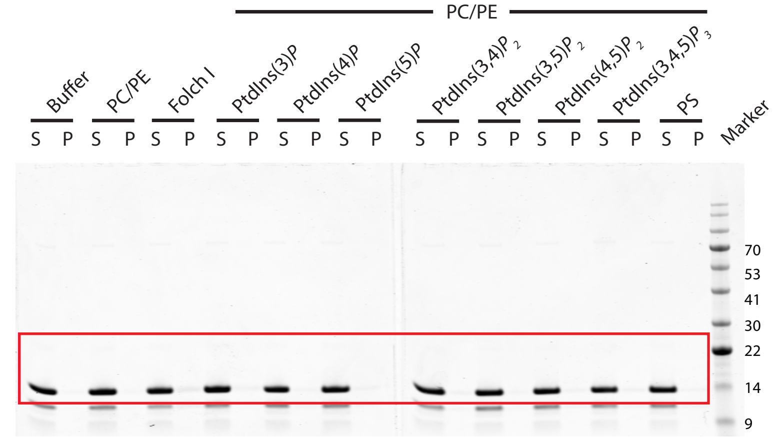

SNX1_Y194A/K196A/K200A

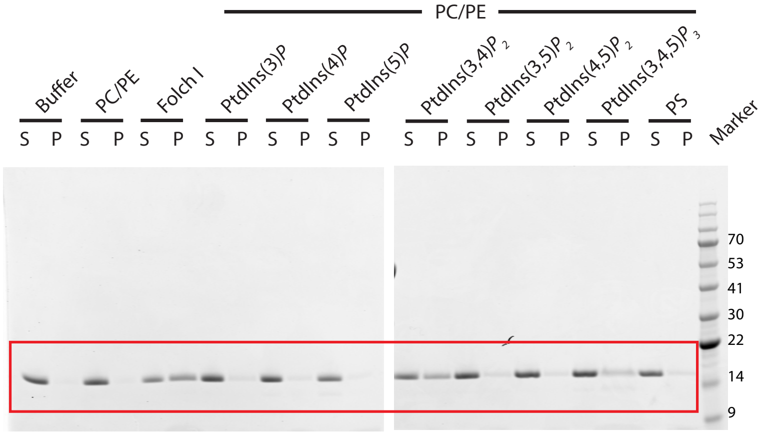

SNX1_K213A

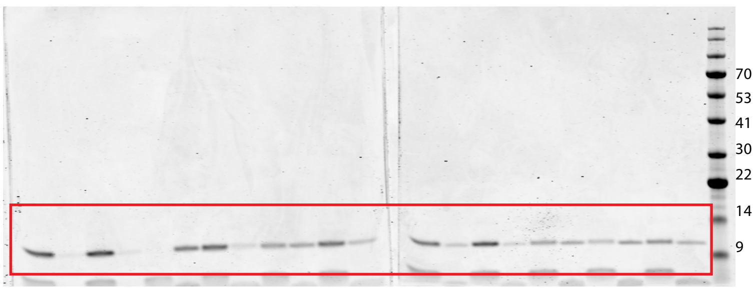

SNX15_H59A

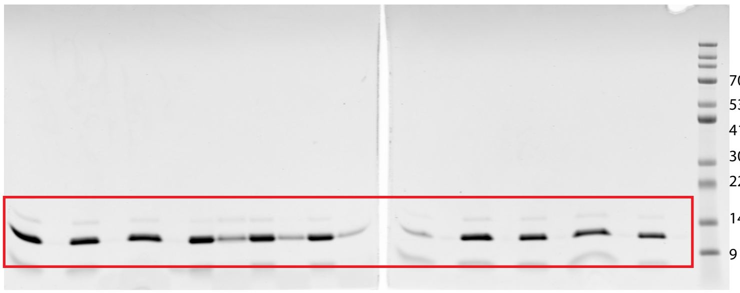

SNX15_Y64A/R67A/R71A/R72A

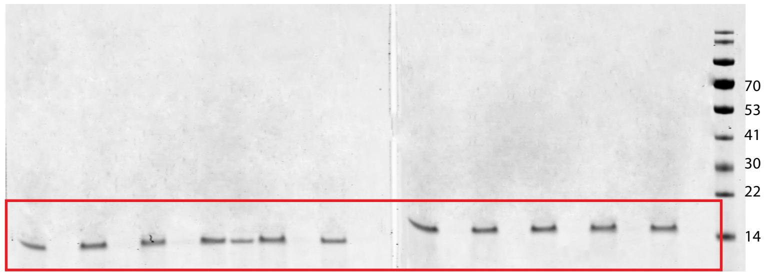

SNX23_H1228A/K1232A/K1234A

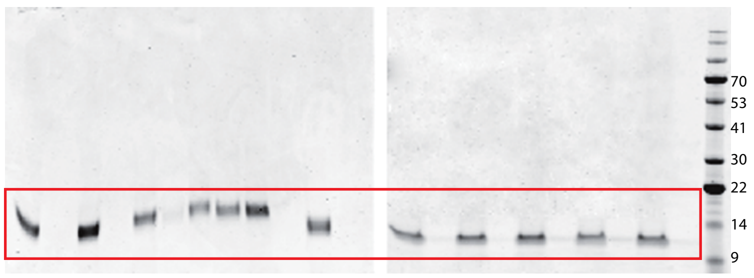

SNX24_H46A/K50A

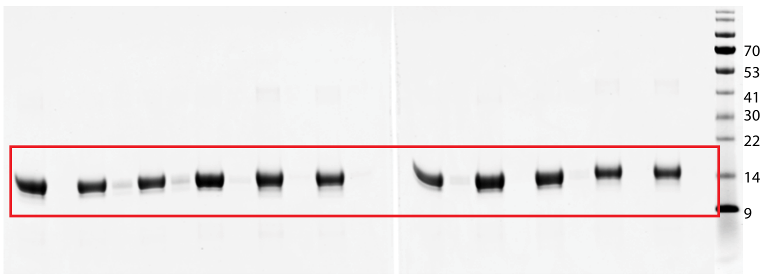

SNX25_H720A

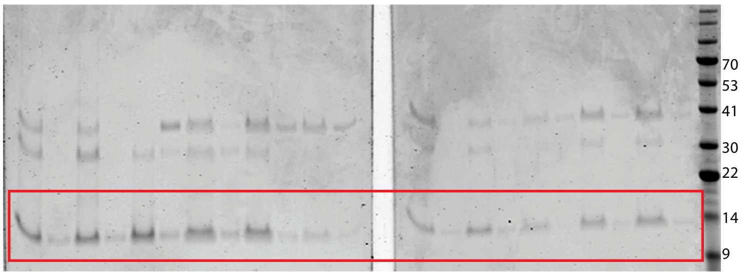

p47phox_H51A/K55A

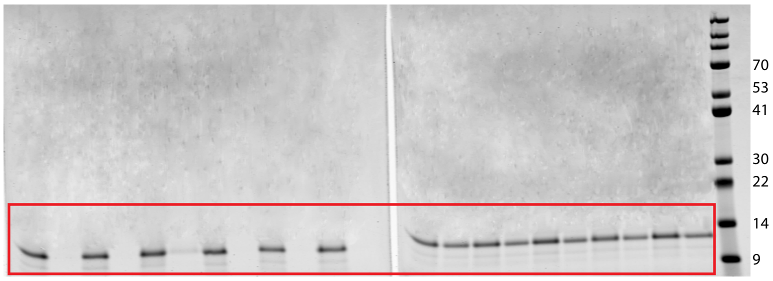

p47phox_R44A

Raw gels related to Figure 7

Supplementary Table 1. Previously reported structures and PtdIns*P*-binding specificities of mammalian PX proteins.

Family	PX protein	Aliases	PDB ID	Structure (method/ligand)	References	PtdIns*P* specificity	Methods	K_d (□M)	References
**PX-BAR**	SNX1		2I4K	NMR/apo	(1)	PI(3,5)P2	PIP strip	-	(2)
						PI(3,4,5)P3	PIP strip	-	(2)
						PI(4,5)P2	PIP strip	-	(3)
						PI(3,4)P2	PIP strip	-	(3)
						PI(3,5)P2	PIP strip	-	(3)
						PI(3,4,5)P3	PIP strip	-	(3)
						PI(3)P	Liposome binding	1.1	(3)
						PI(3,5)P2	Liposome binding	3.1	(3)
						PI3P	Fluorescent liposome binding	0.24	(4)
						PI(3,5)P2	PIP pulldown	-	(5)
	SNX2	TRG-9	-			PI(3)P	PIP strip	-	(2)
						PI(3)P	Liposome binding	4.4	(6)
						PI(3,5)P2	Liposome binding	8.3	(6)
						PI(3,5)P2	PIP pulldown	-	(5)
	SNX4		-			PI3P	Liposome pelleting	-	(7)
	SNX5		3HPC	X-ray/apo	(8)	PI3P	Liposome binding	-	(9)
			3HPB	X-ray/apo	(8)	PI(3,4)P2	Liposome binding	-	(9)
			5WY2	X-ray/IncE peptide	(10)	PI(4,5)P2	NMR titration	410	(8)
			5TGI	X-ray/IncE peptide	(11)	PI(4)P	PIP strip	-	(12)
			5TGJ	X-ray/IncE peptide	(11)	PI(5)P	PIP strip	-	(12)
			5TGH	X-ray/IncE peptide	(11)	PI(3,5)P2	PIP strip	-	(12)
			5TP1	X-ray/IncE peptide	(13)	PI(3,4,5)P3	PIP pulldown	-	(14)
	SNX6	TFAF2	-			PI4P	Liposome pelleting	-	(15)
	SNX7		3IQ2	X-ray/SO_4	-	PI(3)P	Lipid overlay in microtitre plate	-	(16)
	SNX8		-			-			
	SNX30			-		-			
	SNX32		6E8R	X-ray/IncE peptide	-	-			
**SH3-PX-BAR**	SNX9	SH3PX1, SH3PXD3A	3DYU	X-ray/apo	(17)	Generic PtdIns lipids	PIP strip	-	(18)
			3DYT	X-ray/apo	(17)	PI(3)P	Liposome binding	-	(19)
			2RAJ	X-ray/SO_4	(20)	PI(3,4)P2	Liposome binding	-	(19)
			2RAK	X-ray/PI(3)P	(20)	PI(4,5)P2	Liposome binding	-	(19)
			2RAI	X-ray/apo	(20)	PI(3,4,5)P3	Liposome binding	-	(19)
						PI(3,4,5)P3	PIP strip	-	(21)
						PI(4,5)P2	Liposome binding	-	(22)
						PI(3)P	Liposome binding	-	(20)
						PI(4,5)P2	Liposome binding	-	(20)
						PI(3,4)P2	Liposome binding	-	(23)
						PI(4,5)P2	Liposome binding	-	(23)
						PI(3,4,5)P3	Liposome binding	-	(23)
						PI(3,5)P2	Liposome binding	-	(23)
						PI(3,4)P2	PIP strip and liposome binding	-	(24)
						PI(4,5)P2	PIP strip and liposome binding	-	(24)
						PI(3,4,5)P3	PIP strip and liposome binding	-	(24)
						PI(3,5)P2	PIP strip and liposome binding	-	(24)
						PI(3)P	PIP strip and liposome binding	-	(24)
						PI(4)P	PIP strip and liposome binding	-	(24)
						PI(5)P	PIP strip and liposome binding	-	(24)
	SNX18	SH3PXD3B, SNAG1	-			PI(3,4)P2	Liposome binding	-	(25)
						PI(3,5)P2	Liposome binding	-	(25)
						PI(4,5)P2	Liposome binding	-	(25)
						All PtdIns lipid headgroups	PIP strip	-	(26)
	SNX33	SH3PX3, SH3PXD3C, SNX30	4AKV	X-ray/apo	-	-			
**PXA-RGS-PX-PXC**	SNX13	RGSPX1, KIAA0713	-			PI3P	Liposome pelleting	-	(27)
						PI(3)P	PIP strip	-	(28)
						PI(5)P	PIP strip	-	(28)
						PI(3,5)P2	PIP strip	-	(28)
	SNX14		4PQO	X-ray/apo	(27)	No interactions	Liposome pelleting and NMR	-	(27)
			4PQP	X-ray/apo	(27)				
	SNX19	KIAA0254	4P2I	X-ray/apo	(27)	PI3P	Liposome pelleting	-	(27)
			4P2J	X-ray/SO4	(27)				
	SNX25	MSTP043	5WOE	NMR/apo	This study	-			
**PX-FERM-like**	SNX17	KIAA0064	3FOG	X-ray/apo	-	PI(3)P	PIP strip	-	(29)
			3LUI	X-ray/SO_4	(30)	PI(3)P	Liposome binding	-	(31)
						PI(3)P	PIP strip	-	(32)
						PI(3)P	ITC	18	(30)
						PI(3)P	Liposome binding	-	(30)
	SNX27	KIAA0488, My014	4HAS	X-ray	-	PI(3)P	PIP strip	-	(33)
						PI(3)P	ITC	15	(30)
						PI(3)P	PIP strip	-	(34)
						PI(3)P	Liposome binding		(30)
	SNX31		-			-			
**PX-only**	SNX3		2MX3	NMR/apo	-	PI(3)P	PIP strip	-	(2)

			2F0J	X-ray/Vps26-Vps35	(35)	PI(3)P	Liposome binding	-	(20)
			2F0L	X-ray/Vps26-Vps25-Dmt1-II	(35)	PI(3)P	Lipid overlay in microtitre plate	-	(16)
			2F0M	X-ray/Vps26-Vps25-Dmt1-II	(35)	PI(3)P	PIP strip	-	(36)
						PI(3)P	PIP strip	-	(37)
						PI(3)P	PIP strip	-	(38)
						PI3P	Cellular microinjection	-	(1)
						PI3P	Fluorescent liposome binding	0.19	(4)
						PI3P	Liposome binding	-	(12)
						PI3P	Liposome binding	-	(39)
	SNX10		4ON3	X-ray/apo	(40)	-			
			4PZG	X-ray/apo	(40)				
	SNX11		4IKB	X-ray/apo	(41)	PI3P	PIP strip	-	(41)
			4IKD	X-ray/apo	(41)	PI(3,5)P2	PIP strip	-	(41)
	SNX12		2CSK	NMR/apo	-	PI3P	Fluorescent liposome binding	0.13	(4)
						PI3P	Liposome pelleting	-	(42)
	SNX22		2ETT	NMR/apo	(43)	PI(3)P	NMR titration	1100	(43)
	SNX24	SBBI31, UNQ654, PRO1284	-			-			
	HS1BP3		-			PA at 30%	liposome pelleting and PIP strip	-	(44)
						PI3P	liposome pelleting and PIP strip	-	(44)
						PI4P	PIP strip	-	(44)
						PI5P	PIP strip	-	(44)
						PI(3,4)P2	liposome pelleting and PIP strip	-	(44)
						PI(3,5)P2	liposome pelleting and PIP strip	-	(44)
						PI(3,4,5)P3	liposome pelleting and PIP strip	-	(44)
**PX-SH3**	SH3PXD2A	FISH, KIAA0418, SH3MD1, TKS5	-			PI(3)P	PIP strip	-	(45)
						PI(3,4)P2	PIP strip	-	(45)
	SH3PXD2B	FAD49, KIAA1295, TKS4	-			PI(3)P	PIP strip	-	(46)
						PI(3,4)P2	PIP strip	-	(46)
	SNX28	NOXO1, p41NOX, SH3PXD5	2L73	NMR/apo	-	PI(4)P	PIP strip	-	(47, 48)
						PI(5)P	PIP strip	-	(47, 48)
						PI(3,5)P2	PIP strip	-	(47, 48)
						PI(3)P	PIP strip	-	(49)
						PI(4)P	PIP strip	-	(49)
						PI(5)P	PIP strip	-	(49)
						PI(3,5)P2	PIP strip	-	(49)
						PA	PIP strip	-	(49)
	p40phox	NCF4, SH3PXD4	2DYB	X-ray/apo	(50)	PI(3)P	PIP strip and liposome binding	-	(51)
			1H6H	X-ray/PI(3)P	(52)	PI(3)P	Biosensor liposome interaction		(53)
						PI(3)P	Liposome binding	-	(39)
						PI(3)P	Crystal structure and ITC	5.0	(52)
						PI(3)P	Liposome binding and PIP strip	-	(54)
						PI(3)P	Biosensor liposome interaction	0.001-0.1	(55)
						PI(3)P	Liposome binding	-	(50)
	p47phox	NCF1, SH3PXD1A	1KQ6	X-ray/SO_4	-	PI(3,4)P2	PIP strip	-	(47)
			1O7K	X-ray/SO_4	(56)	PI(3,4)P2	PIP strip and liposome binding	-	(51)
			1GD5	NMR/apo	(57)	PI(3,5)P	PIP strip and liposome binding	-	(51)
						PI(3,4)P2	Liposome binding	-	(39)
						PI(3,4)P2	Liposome binding	-	(54)
						PI(3)P	Liposome binding	-	(54)
						PI(3,5)P2	Liposome binding	-	(54)
						PI(3,4)P2	Biosensor liposome interaction	0.0015	(55)
						PI(3,4)P2	Lipsome binding and Biosensor liposome interaction	34-59	(58)
**PX-S/T kinase**	PXK	MONaKA	-			PI(3)P	PIP strip	-	(59)
	RPS6KC1	RPK118, humS6PKh1	-			PI(3)P	PIP strip	-	(60)
	SGK3	CISK, SGKL	1XTE	X-ray/apo	(61)	PI(3,5)P2	PIP strip	-	(62)
			1XTN	X-ray/SO_4	(61)	PI(4,5)P2	PIP strip	-	(62)
						PI(3,4,5)P3	PIP strip	-	(62)
						PI(3)P	PIP strip and liposome binding	-	(63)
						PI3P	Cellular microinjection	-	(1)
						PI(3)P	PIP strip	-	(64)
**PX-SH3-GAP**	SNX26	ARHGAP33, TC-GAP, NOMA-GAP		-		PI(3)P	PIP strip	-	(65)
						PI(4)P	PIP strip	-	(65)
						PI(4,5)P2	PIP strip	-	(65)
						PI(3,4)P2	PIP strip	-	(65)
	PX-RICS	ARHGAP32, GC-GAP, p250GAP,		-		PI(3)P	PIP strip and liposome binding	-	(66)
						PI(4)P	PIP strip and	-	(66)

		p200GAP, Grit					liposome binding		
						PI(4)P	PIP strip and liposome binding	-	(66)
**PX-PI3-kinase**	PI3K-C2□	PIK3C2A	2RED	X-ray/apo	(67)	PI(4,5)P2	Biosensor liposome interaction	0.025	(68)
			2REA	X-ray/apo	(67)	PI(4,5)P2	Liposome binding	-	(69)
			2AR5	X-ray/apo	(67)				
			2IWL	X-ray/SO_4	(68)				
	PI3K-C2□	PIK3C2B				-			
	PI3K-C2□	PIK3C2G	2WWE	X-ray/apo	-	-			
**PX-PH-PLD**	PLD1		-			PI(5)P	PIP strip	-	(70)
						PI(3)P	Biosensor liposome interaction	0.14	(71)
						PI(4)P	Biosensor liposome interaction	1.8	(71)
						PI(5)P	Biosensor liposome interaction	0.22	(71)
						PI(4,5)P2	Biosensor liposome interaction	1.0	(71)
						PI(3,4)P2	Biosensor liposome interaction	5.0	(71)
						PI(3,5)P2	Biosensor liposome interaction	3.2	(71)
						PI(3,4,5)P3	Biosensor liposome interaction	0.018	(71)
						PI(3,4,5)P3	Liposome pelleting	-	(72)
	PLD2		-			No interactions	Liposome binding	-	(72)
**PX-PXB**	SNX20	SLIC-1	-			PI(4)P	PIP strip	-	(73)
						PI(5)P	PIP strip	-	(73)
						PI(3,5)P2	PIP strip	-	(73)
	SNX21		-			-			
**Kinesin-PX**	KIF16B	SNX23	2V14	X-ray/apo	(74)	PI((3)P	Biosensor liposome interaction	0.028	(75)
			6EE0	X-ray/apo	This study	PI(3,4)P2	Biosensor liposome interaction	0.04	(75)
						PI(3,4,5)P3	Biosensor liposome interaction	0.099	(75)
						PI3P	PIP strip	-	(76)
						PI((3)P	Biosensor liposome interaction	0.0027	(74)
						PI(3,4)P2	Biosensor liposome interaction	0.0028	(74)
						PI(3,5)P2	Biosensor liposome interaction	0.3	(74)
						PI(3,4,5)P3	Biosensor liposome interaction	0.1	(74)
**PX-MIT**	SNX15		6ECM	X-ray/SO4	This study	PI3P	Liposome pelleting and PIP strip	-	(77)
			6MBI	X-ray/SO4	This study				
**PX-LRR-IRAS**	Nischarin	IRAS, KIAA0975	3P0C	X-ray/apo	-	PI(3)P	PIP strip	-	(78)
**PX-SNX16**	SNX16		5GW0	X-ray/apo	(79)	PI(3)P	PIP strip	-	(37)
			5GW1	X-ray/apo	(79)	PI(3)P	PIP strip	-	(80)
			2V14	X-ray		PI3P	Fluorescent liposome binding	0.31	(4)
						PI3P	PIP strip and liposome pelleting	-	(79)
**SNX29-PX**	SNX29	RUNDC2A	-			-			
**PX-SNX34**	SNX34	C6ORF145, PXDC1	-			-			

REFERENCES

1. Zhong Q*, et al.* (2005) Determinants of the endosomal localization of sorting nexin 1. *Molecular biology of the cell* 16(4):2049-2057.

2. Zhong Q*, et al.* (2002) Endosomal localization and function of sorting nexin 1. *Proc Natl Acad Sci U S A* 99(10):6767-6772.

3. Cozier GE*, et al.* (2002) The phox homology (PX) domain-dependent, 3-phosphoinositide-mediated association of sorting nexin-1 with an early sorting endosomal compartment is required for its ability to regulate epidermal growth factor receptor degradation. *The Journal of biological chemistry* 277(50):48730-48736.

4. Ceccato L*, et al.* (2016) PLIF: A rapid, accurate method to detect and quantitatively assess protein-lipid interactions. *Science signaling* 9(421):rs2.

5. Catimel B*, et al.* (2008) The PI(3,5)P2 and PI(4,5)P2 interactomes. *J Proteome Res* 7(12):5295-5313.

6. Carlton JG*, et al.* (2005) Sorting nexin-2 is associated with tubular elements of the early endosome, but is not essential for retromer-mediated endosome-to-TGN transport. *Journal of cell science* 118(Pt 19):4527-4539.

7. Traer CJ*, et al.* (2007) SNX4 coordinates endosomal sorting of TfnR with dynein-mediated transport into the endocytic recycling compartment. *Nat Cell Biol* 9(12):1370-1380.

8. Koharudin LM, Furey W, Liu H, Liu YJ, & Gronenborn AM (2009) The phox domain of sorting nexin 5 lacks PTDINS(3)P specificity and preferentially binds to PTDINS(4,5)P2. *The Journal of biological chemistry*.

9. Merino-Trigo A*, et al.* (2004) Sorting nexin 5 is localized to a subdomain of the early endosomes and is recruited to the plasma membrane following EGF stimulation. *Journal of cell science* 117(Pt 26):6413-6424.

10. Sun Q*, et al.* (2017) Structural and functional insights into sorting nexin 5/6 interaction with bacterial effector IncE. *Signal transduction and targeted therapy* 2:17030.

11. Paul B*, et al.* (2017) Structural basis for the hijacking of endosomal sorting nexin proteins by Chlamydia trachomatis. *eLife* 6.

12. Liu H*, et al.* (2006) Inhibitory regulation of EGF receptor degradation by sorting nexin 5. *Biochem Biophys Res Commun* 342(2):537-546.

13. Elwell CA*, et al.* (2017) Chlamydia interfere with an interaction between the mannose-6-phosphate receptor and sorting nexins to counteract host restriction. *eLife* 6.

14. Catimel B*, et al.* (2009) PI(3,4,5)P3 Interactome. *J Proteome Res* 8(7):3712-3726.

15. Niu Y*, et al.* (2013) PtdIns(4)P regulates retromer-motor interaction to facilitate dynein-cargo dissociation at the trans-Golgi network. *Nat Cell Biol* 15(4):417-429.

16. Xu Y, Hortsman H, Seet L, Wong SH, & Hong W (2001) SNX3 regulates endosomal function through its PX-domain-mediated interaction with PtdIns(3)P. *Nat Cell Biol* 3(7):658-666.

17. Wang Q, Kaan HY, Hooda RN, Goh SL, & Sondermann H (2008) Structure and plasticity of Endophilin and Sorting Nexin 9. *Structure* 16(10):1574-1587.

18. MaCaulay SL*, et al.* (2003) Insulin stimulates movement of sorting nexin 9 between cellular compartments: a putative role mediating cell surface receptor expression and insulin action. *Biochem J* 376(Pt 1):123-134.

19. Lundmark R & Carlsson SR (2003) Sorting nexin 9 participates in clathrin-mediated endocytosis through interactions with the core components. *The Journal of biological chemistry* 278(47):46772-46781.

20. Pylypenko O, Lundmark R, Rasmuson E, Carlsson SR, & Rak A (2007) The PX-BAR membrane-remodeling unit of sorting nexin 9. *Embo J* 26(22):4788-4800.

21. Badour K, *et al.* (2007) Interaction of the Wiskott-Aldrich syndrome protein with sorting nexin 9 is required for CD28 endocytosis and cosignaling in T cells. *Proc Natl Acad Sci U S A* 104(5):1593-1598.

22. Yarar D, Waterman-Storer CM, & Schmid SL (2007) SNX9 couples actin assembly to phosphoinositide signals and is required for membrane remodeling during endocytosis. *Dev Cell* 13(1):43-56.

23. Yarar D, Surka MC, Leonard MC, & Schmid SL (2008) SNX9 activities are regulated by multiple phosphoinositides through both PX and BAR domains. *Traffic* 9(1):133-146.

24. Shin N, *et al.* (2008) SNX9 regulates tubular invagination of the plasma membrane through interaction with actin cytoskeleton and dynamin 2. *Journal of cell science* 121(Pt 8):1252-1263.

25. Haberg K, Lundmark R, & Carlsson SR (2008) SNX18 is an SNX9 paralog that acts as a membrane tubulator in AP-1-positive endosomal trafficking. *Journal of cell science* 121(Pt 9):1495-1505.

26. Nakazawa S, *et al.* (2011) Expression of Sorting Nexin 18 (SNX18) Is Dynamically Regulated in Developing Spinal Motor Neurons. *J Histochem Cytochem* 59(2):202-213.

27. Mas C, *et al.* (2014) Structural basis for different phosphoinositide specificities of the PX domains of sorting nexins regulating G-protein signaling. *The Journal of biological chemistry* 289(41):28554-28568.

28. Zheng B, *et al.* (2001) RGS-PX1, a GAP for GalphaS and sorting nexin in vesicular trafficking. *Science* 294(5548):1939-1942.

29. Czubayko M, Knauth P, Schluter T, Florian V, & Bohnensack R (2006) Sorting nexin 17, a non-self-assembling and a PtdIns(3)P high class affinity protein, interacts with the cerebral cavernous malformation related protein KRIT1. *Biochem Biophys Res Commun* 345(3):1264-1272.

30. Ghai R, *et al.* (2011) Phox homology band 4.1/ezrin/radixin/moesin-like proteins function as molecular scaffolds that interact with cargo receptors and Ras GTPases. *Proc Natl Acad Sci U S A* 108(19):7763-7768.

31. Knauth P, *et al.* (2005) Functions of sorting nexin 17 domains and recognition motif for P-selectin trafficking. *J Mol Biol* 347(4):813-825.

32. van Kerkhof P, *et al.* (2005) Sorting nexin 17 facilitates LRP recycling in the early endosome. *EMBO J* 24(16):2851-2861.

33. Lunn ML, *et al.* (2007) A unique sorting nexin regulates trafficking of potassium channels via a PDZ domain interaction. *Nat Neurosci* 10(10):1249-1259.

34. Rincon E, *et al.* (2011) Translocation dynamics of sorting nexin 27 in activated T cells. *Journal of cell science* 124(Pt 5):776-788.

35. Lucas M, *et al.* (2016) Structural Mechanism for Cargo Recognition by the Retromer Complex. *Cell* 167(6):1623-1635 e1614.

36. Mizutani R, *et al.* (2009) Sorting nexin 3, a protein upregulated by lithium, contains a novel phosphatidylinositol-binding sequence and mediates neurite outgrowth in N1E-115 cells. *Cell Signal* 21(11):1586-1594.

37. Hanson BJ & Hong W (2003) Evidence for a role of SNX16 in regulating traffic between the early and later endosomal compartments. *The Journal of biological chemistry* 278(36):34617-34630.

38. Mor A, *et al.* (2009) Phospholipase D1 regulates lymphocyte adhesion via upregulation of Rap1 at the plasma membrane. *Mol Cell Biol* 29(12):3297-3306.

39. Ago T, *et al.* (2001) The PX domain as a novel phosphoinositide- binding module. *Biochem Biophys Res Commun* 287(3):733-738.

40. Xu T, *et al.* (2014) Structure of human SNX10 reveals insights into its role in human autosomal recessive osteopetrosis. *Proteins* 82(12):3483-3489.

41. Xu J, *et al.* (2013) Structure of sorting nexin 11 (SNX11) reveals a novel extended phox homology (PX) domain critical for inhibition of SNX10-induced vacuolation. *The Journal of biological chemistry* 288(23):16598-16605.

42. Pons V, *et al.* (2012) SNX12 role in endosome membrane transport. *PloS one* 7(6):e38949.

43. Song J, Zhao KQ, Newman CL, Vinarov DA, & Markley JL (2007) Solution structure of human sorting nexin 22. *Protein Sci* 16(5):807-814.

44. Holland P, *et al.* (2016) HS1BP3 negatively regulates autophagy by modulation of phosphatidic acid levels. *Nature communications* 7:13889.

45. Abram CL, *et al.* (2003) The adaptor protein fish associates with members of the ADAMs family and localizes to podosomes of Src-transformed cells. *The Journal of biological chemistry* 278(19):16844-16851.

46. Buschman MD, *et al.* (2009) The novel adaptor protein Tks4 (SH3PXD2B) is required for functional podosome formation. *Molecular biology of the cell* 20(5):1302-1311.

47. Cheng G & Lambeth JD (2004) NOXO1, regulation of lipid binding, localization, and activation of Nox1 by the Phox homology (PX) domain. *The Journal of biological chemistry* 279(6):4737-4742.

48. Cheng G & Lambeth JD (2005) Alternative mRNA splice forms of NOXO1: differential tissue expression and regulation of Nox1 and Nox3. *Gene* 356:118-126.

49. Ueyama T, Lekstrom K, Tsujibe S, Saito N, & Leto TL (2007) Subcellular localization and function of alternatively spliced Noxo1 isoforms. *Free Radic Biol Med* 42(2):180-190.

50. Honbou K, *et al.* (2007) Full-length p40phox structure suggests a basis for regulation mechanism of its membrane binding. *EMBO J* 26(4):1176-1186.

51. Kanai F, *et al.* (2001) The PX domains of p47phox and p40phox bind to lipid products of PI(3)K. *Nat Cell Biol* 3(7):675-678.

52. Bravo J, *et al.* (2001) The crystal structure of the PX domain from p40(phox) bound to phosphatidylinositol 3-phosphate. *Mol Cell* 8(4):829-839.

53. Ellson CD, *et al.* (2001) PtdIns(3)P regulates the neutrophil oxidase complex by binding to the PX domain of p40(phox). *Nat Cell Biol* 3(7):679-682.

54. Zhan Y, Virbasius JV, Song X, Pomerleau DP, & Zhou GW (2002) The p40phox and p47phox PX domains of NADPH oxidase target cell membranes via direct and indirect recruitment by phosphoinositides. *The Journal of biological chemistry* 277(6):4512-4518.

55. Stahelin RV, Burian A, Bruzik KS, Murray D, & Cho W (2003) Membrane binding mechanisms of the PX domains of NADPH oxidase p40phox and p47phox. *The Journal of biological chemistry* 278(16):14469-14479.

56. Karathanassis D, *et al.* (2002) Binding of the PX domain of p47(phox) to phosphatidylinositol 3,4-bisphosphate and phosphatidic acid is masked by an intramolecular interaction. *Embo J* 21(19):5057-5068.

57. Hiroaki H, Ago T, Ito T, Sumimoto H, & Kohda D (2001) Solution structure of the PX domain, a target of the SH3 domain. *Nat Struct Biol* 8(6):526-530.

58. Shmelzer Z, *et al.* (2008) Cytosolic phospholipase A2alpha is targeted to the p47phox-PX domain of the assembled NADPH oxidase via a novel binding site in its C2 domain. *The Journal of biological chemistry* 283(46):31898-31908.

59. Takeuchi H, Takeuchi T, Gao J, Cantley LC, & Hirata M (2010) Characterization of PXK as a protein involved in epidermal growth factor receptor trafficking. *Mol Cell Biol.*

60. Hayashi S, *et al.* (2002) Identification and characterization of RPK118, a novel sphingosine kinase-1-binding protein. *The Journal of biological chemistry* 277(36):33319-33324.

61. Xing Y, *et al.* (2004) Structural basis of membrane targeting by the Phox homology domain of cytokine-independent survival kinase (CISK-PX). *The Journal of biological chemistry* 279(29):30662-30669.

62. Xu J, Liu D, Gill G, & Songyang Z (2001) Regulation of cytokine-independent survival kinase (CISK) by the Phox homology domain and phosphoinositides. *J Cell Biol* 154(4):699-705.

63. Virbasius JV, *et al.* (2001) Activation of the Akt-related cytokine-independent survival kinase requires interaction of its phox domain with endosomal phosphatidylinositol 3-phosphate. *Proc Natl Acad Sci U S A* 98(23):12908-12913.

64. Tessier M & Woodgett JR (2006) Role of the Phox homology domain and phosphorylation in activation of serum and glucocorticoid-regulated kinase-3. *The Journal of biological chemistry* 281(33):23978-23989.

65. Chiang SH, *et al.* (2003) TCGAP, a multidomain Rho GTPase-activating protein involved in insulin-stimulated glucose transport. *EMBO J* 22(11):2679-2691.

66. Hayashi T, *et al.* (2007) PX-RICS, a novel splicing variant of RICS, is a main isoform expressed during neural development. *Genes Cells* 12(8):929-939.

67. Parkinson GN, Vines D, Driscoll PC, & Djordjevic S (2008) Crystal structures of PI3K-C2alpha PX domain indicate conformational change associated with ligand binding. *BMC Struct Biol* 8:13.

68. Stahelin RV, *et al.* (2006) Structural and membrane binding analysis of the Phox homology domain of phosphoinositide 3-kinase-C2alpha. *The Journal of biological chemistry* 281(51):39396-39406.

69. Song X, *et al.* (2001) Phox homology domains specifically bind phosphatidylinositol phosphates. *Biochemistry* 40(30):8940-8944.

70. Du G, *et al.* (2003) Regulation of phospholipase D1 subcellular cycling through coordination of multiple membrane association motifs. *J Cell Biol* 162(2):305-315.

71. Stahelin RV, *et al.* (2004) Mechanism of membrane binding of the phospholipase D1 PX domain. *The Journal of biological chemistry* 279(52):54918-54926.

72. Lee JS, *et al.* (2005) Phosphatidylinositol (3,4,5)-trisphosphate specifically interacts with the phox homology domain of phospholipase D1 and stimulates its activity. *Journal of cell science* 118(Pt 19):4405-4413.

73. Schaff UY, *et al.* (2008) SLIC-1/sorting nexin 20: a novel sorting nexin that directs subcellular distribution of PSGL-1. *Eur J Immunol* 38(2):550-564.

74. Blatner NR, *et al.* (2007) The structural basis of novel endosome anchoring activity of KIF16B kinesin. *EMBO J* 26(15):3709-3719.

75. Hoepfner S, *et al.* (2005) Modulation of receptor recycling and degradation by the endosomal kinesin KIF16B. *Cell* 121(3):437-450.

76. Pyrpassopoulos S, Shuman H, & Ostap EM (2017) Adhesion force and attachment lifetime of the KIF16B-PX domain interaction with lipid membranes. *Molecular biology of the cell* 28(23):3315-3322.

77. Danson C, *et al.* (2013) SNX15 links clathrin endocytosis to the PtdIns3P early endosome independently of the APPL1 endosome. *Journal of cell science* 126(Pt 21):4885-4899.

78. Lim KP & Hong W (2004) Human Nischarin/imidazoline receptor antisera-selected protein is targeted to the endosomes by a combined action of a PX domain and a coiled-coil region. *The Journal of biological chemistry* 279(52):54770-54782.

79. Xu J, *et al.* (2017) SNX16 Regulates the Recycling of E-Cadherin through a Unique Mechanism of Coordinated Membrane and Cargo Binding. *Structure* 25(8):1251-1263 e1255.

80. Choi JH, *et al.* (2004) Sorting nexin 16 regulates EGF receptor trafficking by phosphatidylinositol-3-phosphate interaction with the Phox domain. *Journal of cell science* 117(Pt 18):4209-4218.

Supplementary Table 2. PX domain constructs used in this study.

PX domain	Sequence	Cloned into pGEX4T-2	Successful purification
HS1BP3 (17-139)	TGLDLTVPQHQEVRGKMMSGHVEYQILVV TRLAAFKSAKHRPEDVVQFLVSKKYSEIEEF YQKLSSRYAAASLPPLPRKVLFVGESDIRER RAVFNEILRCVSKDAELAGSPELLEFLGTRS	Yes	Yes
IRAS (13-124)	AEPAKEARVVGSELVDTYTVYIIQVTDGSHE WTVKHRYSDFHDLHEKLVAERKIDKNLLPP KKIIGKNSRSLVEKREKDLEVYLQKLLAAFP GVTPRVLAHFLHFHFYEING	Yes	Yes
p40phox (1-144)	MAVAQQLRAESDFEQLPDDVAISANIADIEE KRGFTSHFVFVIEVKTKGGSKYLIYRRYRQF HALQSKLEERFGPDSKSSALACTLPTLPAKV YVGVKQEIAEMRIPALNAYMKSLLSLPVWV LMDEDVRIFFYQSPYDSEQVP	Yes	Yes
p47phox (1-122)	MGDTFIRHIALLGFEKRFVPSQHYVYMFLV KWQDLSEKVVYRRFTEIYEFHKTLKEMFPIE AGAINPENRIIPHLPAPKWFDGQRAAENRQG TLTEYCGTLMSLPTKISRCPHLLDFFKVRP	Yes	Yes
PI3KC2A (1405-1545)	DEPILSFSPKTYSFRQDGRIKEVSVFTYHKKY NPDKHYIYVVRILREGQIEPSFVFRTFDEFQE LHNKLSIIFPLWKLPGFPNRMVLGRTHIKDV AAKRKIELNSYLQSLMNASTDVAECDLVCT FFHPLLRDEKAEGIA	Yes	Yes
PI3KC2B (1348-1487)	DRLTLSFASRTHTLKSSGRISDVFLCRHEKIF HPNKGYIYVVKVMRENTHEATYIQRTFEEF QELHNKLRLLFPSSHLPSFPSRFVIGRSRGEA VAERRREELNGYIWHLIHAPPEVAECDLVY TFFHPLPRDEKAMGTS	Yes	Yes
PI3KC2G (1200-1310)	STTRSIERATILGFSKKSSNLYLIQVTHSNNE TSLTEKSFEQFSKLHSQLQKQFASLTLPEFPH WWHLPFTNSDHRRFRDLNHYMEQILNVSH EVTNSDCVLSFFLSEAVQ	Yes	Yes
PLD1 (80-211)	PIKAQVLEVERFTSTTRVPSINLYTIELTHGE FKWQVKRKFKHFQEFHRELLKYKAFIRIPIP TRRHTFRRQNVREEPREMPSLPRSSENMIRE EQFLGRRKQLEDYLTKILKMPMYRNYHATT EFLDISQL	Yes	No
PLD2 (61-191)	PGVPVTAQVVGTERYTSGSKVGTCTLYSVR LTHGDFSWTTKKKYRHFQELHRDLLRHKV LMSLLPLARFAVAYSPARDAGNREMPSLPR AGPEGSTRHAASKQKYLENYLNRLLTMSFY RNYHAMTEFLEV	Yes	No
PXK (1-125)	MAFMEKPPAGKVLLDDTVPLTAAIEASQSL QSHTEYIIRVQRGISVENSWQIVRRYSDFDLL NNSLQIAGLSLPLPPKKLIGNMDREFIAERQ KGLQNYLNVITTNHILSNCELVKKFLDPNNY S	Yes	Yes
PXRICS (127-255)	NVEFGSIQLSLSEEQNEVMKNGCESKELVYL VQIACQGKSWIVKRSYEDFRVLDKHLHLCI YDRRFSQLSELPRSDTLKDSPESVTQMLMA YLSRLSAIAGNKINCGPALTWMEIDNKGNH LLVHEESS	Yes	Yes
RPS6KC1 (1-141)	MTSYRERSADLARFYTVTEPQRHPRGYTVY KVTARVVSRRNPEDVQEIIVWKRYSDFKKL HKELWQIHKNLFRHSELFPPFAKGIVFGRFD	Yes	Yes

	ETVIEERRQCAEDLLQFSANIPALYNSKQLE DFFKGGIINDSSELIGPAE		
SGK3 (1-126)	MQRDHTMDYKESCPSVSIPSSDEHREKKKR FTVYKVLVSVGRSEWFVFRRYAEFDKLYNT LKKQFPAMALKIPAKRIFGDNFDPDFIKQRR AGLNEFIQNLVRYPELYNHPDVRAFLQMDS PKHQS	Yes	Yes
SH3PXD2A (1-126)	MLAYCVQDATVVDVEKRRNPSKHYVYIIN VTWSDSTSQTIYRRYSKFFDLQMQLLDKFPI EGGQKDPKQRIIPFLPGKILFRRSHIRDVAVK RLKPIDEYCRALVRLPPHISQCDEVFRFFEAR P	Yes	Yes
SH3PXD2B (1-125)	MPPRRSIVEVKVLDVQKRRVPNKHYVYIIR VTWSSGSTEAIYRRYSKFFDLQMQMLDKFP MEGGQKDPKQRIIPFLPGKILFRRSHIRDVAV KRLIPIDEYCKALIQLPPYISQCDEVLQFFET	No	No
SNX1 (142-269)	QFDLTVGITDPEKIGDGMNAYVAYKVTTQT SLPLFRSKQFAVKRRFSDFLGLYEKLSEKHS QNGFIVPPPPEKSLIGMTKVKVGKEDSSSAE FLEKRRAALERYLQRIVNHPTMLQDPDVRE FLEKEE	Yes	Yes
SNX10 (1-201)	MFPEQQKEEFVSVWVRDPRIQKEDFWHSYI DYEICIHTNSMCFTMKTSCVRRRYREFVWL RQRLQSNALLVQLPELPSKNLFFNMNNRQH VDQRRQGLEDFLRKVLQNALLLSDSSLHLF LQSHLNSEDIEACVSGQTKYSVEEAIHKFAL MNRRFPEEDEEGKKENDIDYDSESSSSGLGH SSDDSSSHGCKVNTAPQES	Yes	Yes
SNX11 (7-167)	MSENQEQEEVITVRVQDPRVQNEGSWNSY VDYKIFLHTNSKAFTAKTSCVRRRYREFVW LRKQLQRNAGLVPVPELPGKSTFFGTSDEFI EKRRQGLQHFLEKVLQSVVLLSDSQLHLFL QSQLSVPEIEACVQGRSTMTVSDAILRYAMS NCGWAQEERQ	Yes	Yes
SNX12 (1-162)	MSDTAVADTRRLNSKPQDLTDAYGPPSNFL EIDIFNPQTVGVGRARFTTYEVRMRTNLPIF KLKESCVRRRYSDFEWLKNELERDSKIVVPP LPGKALKRQLPFRGDEGIFEESFIEERRQGLE QFINKIAGHPLAQNERCLHMFLQEEAIDRNY VPGKVRQ	Yes	Yes
SNX13 (1-100)	SDDSVQLHAYISDTGVCNDHGKTYALYAIT VHRRNLNSEEMWKTYRRYSDFHDFHMRIT EQFESLSSILKLPGKKTFNNMDRDFLEKRKK DLNAYLQLLLAPEMMKASPALAHYVYDFL ENK	Yes	Yes
SNX14 (561-686)	NLAAWKISIPYVDFFEDPSSERKEKKERIPVF CIDVERNDRRAVGHEPEHWSVYRRYLEFYV LESKLTEFHGAFPDAQLPSKRIIGPKNYEFLK SKREEFQEYLQKLLQHPELSNSQLLADFLSP N	Yes	Yes
SNX15 (1-127)	MSRQAKDDFLRHYTVSDPRTHPKGYTEYK VTAQFISKKDPEDVKEVVVWKRYSDFRKLH GDLAYTHRNLFRRLEEFPAFPRAQVFGRFEA SVIEERRKGAEDLLRFTVHIPALNNSPQLKEF FRGG	Yes	Yes
SNX16 (104-216)	EDRPSTPTILGYEVMEERAKFTVYKILVKKT PEESWVVFRRYTDFSRLNDKLKEMFPGFRL ALPPKRWFKDNYNADFLEDRQLGLQAFLQ NLVAHKDIANCLAVREFLCLDDP	Yes	Yes

SNX17<br>(1-111)	MHFSIPETESRSGDSGGSAYVAYNIHVNGVL<br>HCRVRYSQLLGLHEQLRKEYGANVLPAFPP<br>KKLFSLTPAEVEQRREQLEKYMQAVRQDPL<br>LGSSETFNSFLRRAQQETQQ	Yes	Yes
SNX18<br>(271-403)	ENPYPFQCTIDDPTKQTKFKGMKSYISYKLV<br>PTHTQVPVHRRYKHFDWLYARLAEKFPVIS<br>VPHLPEKQATGRFEEDFISKRRKGLIWWMN<br>HMASHPVLAQCDVFQHFLTCPSSTDEKAW<br>KQGKRKAEKDEMV	Yes	No
SNX19<br>(1-100)	NLRITGTITAREHSGTGFHPYTLYTVKYETA<br>LDGENSSGLQQLAYHTVNRRYREFLNLQTR<br>LEEKPDLRKFIKNVKGPKKLFPDLPFGNMDS<br>DRVEARKSLLESFLKQLCAIPEIANSEEVQEF<br>LAL	Yes	Yes
SNX2<br>(136-279)	NGDIFDIEIGVSDPEKVGDGMNAYMAYRVT<br>TKTSLSMFSKSEFSVKRRFSDFLGLHSKLAS<br>KYLHVGYIVPPAPEKSIVGMTKVKVGKEDS<br>SSTEFVEKRRAALERYLQRTVKHPTLLQDP<br>DLRQFLESSELPRAVNTQALSGA	Yes	Yes
SNX20<br>(71-184)	WKHVKLLFEIASARIEERKVSKFVVYQIIVIQ<br>TGSFDNNKAVLERRYSDFAKLQKALLKTFR<br>EEIEDVEFPRKHLTGNFAEEMICERRRALQE<br>YLGLLYAIRCVRRSREFLDFL	Yes	No
SNX21<br>(130-251)	RLLFEVTSANVVKDPPSKYVLYTLAVIGPGP<br>PDCQPAQISRRYSDFERLHRNLQRQFRGPM<br>AAISFPRKRLRRNFTAETIARRSRAFEQFLGH<br>LQAVPELRHAPDLQDFFVLPELRRAQSLT	Yes	No
SNX22<br>(1-120)	MLEVHIPSVGPEAEGPRQSPEKSHMVFRVE<br>VLCSGRRHTVPRRYSEFHALHKRIKKLYKV<br>PDFPSKRLPNWRTRGLEQRRQGLEAYIQGIL<br>YLNQEVPKELLEFLRLRHFPTDPKASNWG	Yes	Yes
SNX23<br>(1178-1312)	DLKDPIKISIPRYVLCGQGKDAHFEFEVKITV<br>LDETWTVFRRYSRFREMHKTLKLKYAELA<br>ALEFPPKKLFGNKDERVIAERRSHLEKYLRD<br>FFSVMLQSATSPLHINKVGLTLSKHTICEFSP<br>FFKKGVFDYS	Yes	Yes
SNX24<br>(1-101)	MEVYIPSFRYEESDLERGYTVFKIEVLMNGR<br>KHFVEKRYSEFHALHKKLKKCIKTPEIPSKH<br>VRNWVPKVLEQRRQGLETYLQAVILENEEL<br>PKLFLDFLN	Yes	Yes
SNX25<br>(506-628)	NLGMWKASITSGEVTEENGEQLPCYFVMVS<br>LQEVGGVETKNWTVPRRLSEFQNLHRKLSE<br>CVPSLKKVQLPSLSKLPFKSIDQKFMEKSKN<br>QLNKFLQNLLSDERLCQSEALYAFLSPSPDY<br>L	Yes	Yes
SNX26<br>(56-172)	NVDFGHIQLLLSPDREGPSLSGENELVFGVQ<br>VTCQGRSWPVLRSYDDFRSLDAHLHRCIFD<br>RRFSCLPELPPPPEGARAAQMLVPLLLQYLE<br>TLSGLVDSNLNCGPVLTWMELDNHG	Yes	Yes
SNX27<br>(156-265)	DYTEKQAVPISVPRYKHVEQNGEKFVVYNV<br>YMAGRQLCSKRYREFAILHQNLKREFANFT<br>FPRLPGKWPFSLSEQQLDARRRGLEEYLEK<br>VCSIRVIGESDIMQEFLSES	Yes	Yes
SNX28<br>(1-124)	MAGPRYPVSVQGAALVQIKRLQTFAFSVRW<br>SDGSDTFVRRSWDEFRQLKTLKETFPVEAG<br>LLRRSDRVLPKLLDAPLLGRVGRTSRGLAR<br>LQLLETYSRRLLATAERVARSPTITGFFAPQP<br>LD	Yes	Yes

SNX29 (269-391)	NRALINVWIPSVFLRGKAANAFHVYQVYIRI KDDEWNIYRRYTEFRSLHHKLQNKYPQVR AYNFPPKKAIGNKDAKFVEERRKQLQNYLR SVMNKVIQMVPEFAASPKKETLIQLMPFFV DIT	Yes	Yes
SNX3 (21-151)	AYGPPSNFLEIDVSNPQTVGVGRGRFTTYEI RVKTNLPIFKLKESTVRRRYSDFEWLRSELE RESKVVVPPLPGKAFLRQLPFRGDDGIFDDN FIEERKQGLEQFINKVAGHPLAQNERCLHMF LQDEIID	Yes	Yes
SNX30 (86-205)	GETRDLFVIVDDPKKHVCTMETYITYRITTK STRVEFDLPEYSVRRRYQDFDWLRSKLEES QPTHLIPPLPEKFVVKGVVDRFSEEFVETRR KALDKFLKRITDHPVLSFNEHFNIFLTA	No	No
SNX31 (1-106)	MKMHFCIPVSQQRSDALGGRYVLYSVHLD GFLFCRVRYSQLHGWNEQLRRVFGNCLPPF PPKYYLAMTTAMAHERRDQLEQYLQNVTM DPNVLRSDVFVEFLKLAQ	Yes	Yes
SNX32 (17-166)	SVDLQGDSSLQVEISDAVSERDKVKFTVQT KSCLPHFAQTEFSVVRQHEEFIWLHDAYVE NEEYAGLIIPPAPPRPDFEASREKLQKLGEGD SSVTREEFAKMKQELEAEYLAIFKKTVAMH EVFLQRLAAHPTLRRDHNFFVFLEYGQD	Yes	Yes
SNX33 (220-364)	GPQWKANPHPFACSVEDPTKQTKFKGIKSYI SYKLTPTHAASPVYRRYKHFDWLYNRLLH KFTVISVPHLPEKQATGRFEEDFIEKRKRRLI LWMDHMTSHPVLSQYEGFQHFLSCLDDKQ WKMGKRRAEKDEMVGASFLLTFQI	No	No
SNX34 (1-130)	MASAVFEGTSLVNMFVRGCWVNGIRRLIVS RRGDEEEFFEIRTEWSDRSVLYLHRSLADLG RLWQRLRDAFPEDRSELAQGPLRQGLVAIK EAHDIETRLNEVEKLLKTIISMPCKYSRSEVV LTFFERS	Yes	No
SNX4 (48-186)	MTHNNFWLKKIEISVSEAEKRTGRNAMNM QETYTAYLIETRSVEHTDGQSVLTDSLWRR YSEFELLRSYLLVYYPHIVVPPLPEKRAEFV WHKLSADNMDPDFVERRRIGLENFLLRIAS HPILCRDKIFYLFLTQEGN	Yes	Yes
SNX5 (26-170)	SVDLNVDPSLQIDIPDALSERDKVKFTVHTK TTLPTFQSPEFSVTRQHEDFVWLHDTLIETT DYAGLIIPPAPTKPDFDGPREKMQKLGEGEG SMTKEEFAKMKQELEAEYLAVFKKTVSSHE VFLQRLSSHPVLSKDRNFHVFLEYDQ	Yes	Yes
SNX6 (29-170)	QSDAALQVDISDALSERDKVKFTVHTKSSLP NFKQNEFSVVRQHEEFIWLHDSFVENEDYA GYIIPPAPPRPDFDASREKLQKLGEGEGSMT KEEFTKMKQELEAEYLAIFKKTVAMHEVFL CRVAAHPILRRDLNFHVFLEYNQ	Yes	Yes
SNX7 (89-218)	DEPDLKDLFITVDEPESHVTTIETFITYRIITK TSRGEFDSSEFEVRRRYQDFLWLKGKLEEA HPTLIIPPLPEKFIVKGMVERFNDDFIETRRK ALHKFLNRIADHPTLTFNEDFKIFLTAQAWE LSSH	Yes	Yes
SNX8 (70-190)	ELLARDTVQVELIPEKKGLFLKHVEYEVSSQ RFKSSVYRRYNDFVVFQEMLLHKFPYRMVP ALPPKRMLGADREFIEARRRALKRFVNLVA RHPLFSEDVVLKLFLSFSGSDVQNKLKESA	Yes	No
SNX9 (246-376)	PTSTFDCVVADPRKGSKMYGLKSYIEYQLT PTNTNRSVNHRYKHFDWLYERLLVKFGSAI PIPSLPDKQVTGRFEEEFIKMRMERLQAWM	Yes	Yes

	TRMCRHPVISESEVFQQFLNFRDEKEWKTG<br>KRKAERDELAG		
		46/49	39/49

Supplementary Table 3. Primers used in this study for site-directed mutagenesis of PX domains

Mutation	Forward Primer	Reverse Primer
SNX1 Y194A/K196A/K200A	CTGGGTCTGGCCGAGGCGCTGAGCGAAGCACACAGCCAA	TTGGCTGTGTGCTTCGCTCAGCGCCTCGGCCAGACCCAG
SNX1 K213A	CCGCCGCCGCCGGAGGCGAGCCTGATTGGTATG	CATACCAATCAGGCTCGCCTCCGGCGGCGGCGG
SNX2 H187A/K189A/K193A	CTGGGTCTGGCCAGCGCGCTGGCGAGCGCATACCTGCAC	GTGCAGGTATGCGCTCGCCAGCGCGCTGGCCAGACCCAG
SNX9 Y290A/R292A/K296A	GATTGGCTGGCCGAAGCTCTGCTGGTGGCGTTTGGCAGT	ACTGCCAAACGCCACCAGCAGAGCTTCGGCCAGCCAATC
SNX15 H59A	GATTTTCGCAAACTGGCTGGCGACCTGGCCTAC	GTAGGCCAGGTCGCCAGCCAGTTTGCGAAAATC
SNX15 Y64A/R67A/R71A/R72A	CTGGCCGCCACGCACGCTAACCTGTTCGCTGCCCTGGAA	TTCCAGGGCAGCGAACAGGTTAGCGTGCGTGGCGGCCAG
SNX23 H1224A/K1228A/K1230A	CGCGAAATGGCTAAAACGCTGGCACTGGCGTACGCCGAA	TTCGGCGTACGCCAGTGCCAGCGTTTTAGCCATTTCGCG
SNX24 H46A/K50A	GAATTTCACGCGCTGGCCAAGAAACTGGCGAAATGCATCAAG	CTTGATGCATTTCGCCAGTTTCTTGGCCAGCGCGTGAAATTC
SNX25 H720A	CAGAACCTGGCTCGCAAACTG	CAGTTTGCGAGCCAGGTTCTG
SNX25 H558A/K560A	TTTCAAAATCTGGCTCGTGCACTGTCAGAATGC	GCATTCTGACAGTGCACGAGCCAGATTTTGAAA
SNX29 H315A/K317A/K321A	CGTAGTCTGGCTCACGCACTGCAGAACGCGTATCCGCAA	TTGCGGATACGCGTTCTGCAGTGCGTGAGCCAGACTACG
p47phox R44A	GTGGTTTATCGTGCCTTCACCGAAATC	GATTTCGGTGAAGGCACGATAAACCAC
p47phox	GAAATCTACGAATTTGCTAAGACGCTGGCGGAAATGTTTCCG	CGGAAACATTTCCGCCAGCGTCTTAGCAAATTCGTAGATTTC
SGK3 Y47A/K51A/K52A	GATAAGCTGGCCAACACGCTGGCAGCGCAGTTTCCG	CGGAAACTGCGCTGCCAGCGTGTTGGCCAGCTTATC
PI3KC2α H1453A/K1455A	CAAGAACTGGCTAACGCACTGTCCATT	AATGGACAGTGCGTTAGCCAGTTCTTG

Supplementary Table 4. ITC binding parameters measured for selected PX domains[a]

	Cell Conc. (µM)	Syr. Conc. (µM)	K_d (µM)	ΔH (kcal/mol)	ΔG (kcal/mol)	-TΔS (kcal/mol)	N
**PX + PtdIns3*P***							
SNX1 + PtdIns3*P*	20	500	NB	NB	NB	NB	NB
SNX2 + PtdIns3*P*	20	500	NB	NB	NB	NB	NB
SNX3 + PtdIns3*P*	20	500	15.0 ± 2.24	-10.7 ± 0.75	-6.58	4.11	1.40 ± 0.12
SNX4 + PtdIns3*P*	20	500	28.8 ± 2.16	-3.53 ± 0.13	-5.97	-2.47	0.87 ± 0.09
SNX5 + PtdIns3*P*	20	500	NB	NB	NB	NB	NB
SNX6 + PtdIns3*P*	20	500	NB	NB	NB	NB	NB
SNX7 + PtdIns3*P*	20	500	14.2 ± 2.14	-12.6 ± 1.12	-6.62	6.02	1.25 ± 0.13
SNX9 + PtdIns3*P*	20	500	1.56 ± 0.25	-15.0 ± 2.31	-7.92	-7.09	0.75 ± 0.11
SNX10 + PtdIns3*P*	20	500	31.8 ± 2.25	-4.35 ± 0.31	-5.63	-1.93	0.80 ± 0.07
SNX11 + PtdIns3*P*	20	500	4.30 ± 0.67	-5.99 ± 0.34	-7.32	-1.33	0.93 ± 0.10
SNX12 + PtdIns3*P*	20	500	24.6 ± 5.10	-19.7 ± 7.73	-6.29	13.4	1.03 ± 0.19
SNX13 + PtdIns3*P*	20	500	15.6 ± 4.37	-15.7 ± 4.36	-6.56	9.13	0.75 ± 0.15
SNX14 + PtdIns3*P*	20	500	NB	NB	NB	NB	NB
SNX15 + PtdIns3*P*	20	500	11.9 ± 2.2	-1.81 ± 0.5	-0.72	-4.92	1.15 ± 0.10
SNX16 + PtdIns3*P*	20	500	16.3 ± 2.13	-2.62 ± 0.23	-6.61	-3.57	0.89 ± 0.07
SNX17 + PtdIns3*P*	20	500	12.7 ± 4.06	-1.11 ± 0.17	-6.68	-5.57	1.00 ± 0.11
SNX19 + PtdIns3*P*	20	500	13.0 ± 2.80	-10.2 ± 0.98	-6.67	3.54	1.5 ± 0.17
SNX22 + PtdIns3*P*	20	500	16.5 ± 2.76	-16.0 ± 2.14	-6.53	9.51	0.96 ± 0.11
SNX23 + PtdIns3*P*	20	500	0.79 ± 0.40	1.77 ± 0.12	-8.45	-10.2	1.30 ± 0.15
SNX24 + PtdIns3*P*	20	500	2.41 ± 0.50	-1.42 ± 0.85	-7.67	-6.25	1.00 ± 0.10
SNX25 + PtdIns3*P*	20	500	NB	NB	NB	NB	NB
SNX27 + PtdIns3*P*	20	500	11.4 ± 2.27	-10.1 ± 0.85	-6.75	3.31	1.54 ± 0.08
SNX29 + PtdIns3*P*	20	500	2.70 ± 0.20	-8.03 ± 0.21	-7.60	0.43	1.03 ± 0.05
SNX31 + PtdIns3*P*	20	500	12.7 ± 4.06	-1.11 ± 0.17	-6.68	-5.57	1.00 ± 0.08
SNX32 + PtdIns3*P*	20	500	NB	NB	NB	NB	NB
SGK3 + PtdIns3*P*	20	500	1.74 ± 0.11	-14.8 ± 0.17	-7.86	6.96	1.35 ± 0.11
PI3KC2α + PtdIns3*P*	20	500	NB	NB	NB	NB	NB
PI3KC2β + PtdIns3*P*	20	500	NB	NB	NB	NB	NB
PI3KC2γ + PtdIns3*P*	20	500	NB	NB	NB	NB	NB
p40phox + PtdIns3*P*	20	500	9.72 ± 1.66	-9.60 ± 1.09	-6.84	-2.76	0.85 ± 0.15
p47phox + PtdIns3*P*	20	500	11.7 ± 1.82	-5.60 ± 1.09	-6.28	-2.83	0.90 ± 0.13

IRAS + PtdIns3*P*	20	500	2.60 ± 0.65	-9.2 ± 1.38	-7.62	1.55	0.65 ± 0.11
RPS6KC1 + PtdIns3*P*	20	500	1.91 ± 0.43	-0.55 ± 0.03	-7.80	-7.25	0.75 ± 0.09
SH3PXD2A + PtdIns3*P*	20	500	35.0 ± 3.60	-4.11 ± 0.39	-5.36	-1.81	0.88 ± 0.10
PXK + PtdIns3*P*	20	500	13.0 ± 2.80	-10.2 ± 0.98	-6.67	3.54	1.5 ± 0.07
RICS + PtdIns3*P*	20	500	25.7 ± 8.94	-3.38 ± 1.12	-6.25	-3.29	1.12 ± 0.11

**PX + PtdIns(3,4)P_2**							
SNX1 + PtdIns(3,4)P_2	20	500	5.61 ± 0.98	-1.22 ± 0.11	-7.16	-5.94	0.96 ± 0.08
SNX2 + PtdIns(3,4)P_2	20	500	6.35 ± 1.27	-2.80 ± 0.8	-7.09	-4.30	1.00 ± 0.10
SNX9 + PtdIns(3,4)P_2	20	500	9.6 ± 2.5	-3.76 ± 0.26	-6.61	-3.67	0.87 ± 0.10
SNX13 + PtdIns(3,4)P_2	20	500	16.5 ± 2.76	-16.0 ± 2.14	-6.53	9.51	0.96 ± 0.10
SNX15 + PtdIns(3,4)P_2	20	500	2.13 ± 0.67	-2.61 ± 0.28	-7.74	-5.13	0.85 ± 0.11
SNX17 + PtdIns(3,4)P_2	20	500	NB	NB	NB	NB	NB
SNX23 + PtdIns(3,4)P_2	20	500	1.23 ± 0.69	-1.66 ± 0.17	-8.06	-6.40	0.85 ± 0.09
SNX24 + PtdIns(3,4)P_2	20	500	4.24 ± 1.11	-3.88 ± 0.25	-7.33	-3.45	1.51 ± 0.07
SNX25 + PtdIns(3,4)P_2	20	500	304 ± 10.6	-80 ± 5.28	-4.56	75.4	0.65 ± 0.14
SNX27 + PtdIns(3,4)P_2	20	500	NB	NB	NB	NB	NB
SNX29 + PtdIns(3,4)P_2	20	500	4.45 ± 0.74	-6.22 ± 0.55	-7.30	-1.08	0.65 ± 0.09
SGK3 + PtdIns(3,4)P_2	20	500	13.2 ± 4.5	-6.94 ± 2.74	-6.66	0.28	0.85 ± 0.12
PI3KC2α + PtdIns(3,4)P_2	20	500	8.79 ± 1.45	-0.56 ± 0.13	-6.90	-6.34	0.65 ± 0.09
p47phox + PtdIns(3,4)P_2	20	500	22.7 ± 8.94	-3.31 ± 1.02	-6.34	-3.03	1.22 ± 0.11
IRAS + PtdIns(3,4)P_2	20	500	3.15 ± 0.95	-0.63 ± 0.05	-7.51	-6.88	1.04 ± 0.10
RPS6KC1 + PtdIns(3,4)P_2	20	500	1.06 ± 0.20	-2.55 ± 0.12	-8.15	-5.60	0.80 ± 0.06

**PX + PtdIns(3,5)P_2**							
SNX1 + PtdIns(3,5)P_2	20	500	42.0 ± 3.70	-4.08 ± 0.33	-5.97	-1.89	0.78 ± 0.08
SNX2 + PtdIns(3,5)P_2	20	500	35.8 ± 3.36	-3.73 ± 0.27	-6.07	-2.33	0.82 ± 0.10
SNX23 + PtdIns(3,5)P_2	20	500	11.5 ± 2.76	-16.0 ± 2.14	-6.53	9.51	0.96 ± 0.12
SNX25 + PtdIns(3,5)P_2	20	500	12.1 ± 1.25	-16.8 ± 1.29	-6.38	10.5	0.85 ± 0.11

**PX + PtdIns(4,5)P_2**							
SNX1 + PtdIns(4,5)P_2	20	500	11.4 ± 2.34	-4.46 ± 0.42	-6.75	-2.28	1.00 ± 0.09
SNX2 + PtdIns(4,5)P_2	20	500	13.6 ± 2.5	-2.76 ± 0.26	-6.64	-3.88	0.85 ± 0.10
SNX5 + PtdIns(4,5)P_2	20	500	NB	NB	NB	NB	NB

SNX6 + PtdIns(4,5)P_2	20	500	NB	NB	NB	NB	NB
SNX23 + PtdIns(4,5)P_2	20	500	1.90 ± 0.50	-6.73 ± 0.70	-7.81	-1.09	0.76 ± 0.10
SNX25 + PtdIns(4,5)P_2	20	500	2.52 ± 1.11	-6.85 ± 0.80	-7.26	-0.40	0.65 ± 0.09

**PX + PtdIns(3,4,5)P_3**							
SNX1 + PtdIns(3,4,5)P_3	20	500	26.5 ± 7.30	-3.28 ± 0.19	-6.57	-4.04	0.91 ± 0.12
SNX2 + PtdIns(3,4,5)P_3	20	500	20.5 ± 3.6	-2.88 ± 0.36	-6.40	-3.52	0.92 ± 0.09
SNX23 + PtdIns(3,4,5)P_3	20	500	5.20 ± 2.00	-51.1 ± 7.10	-7.22	43.90	0.85 ± 0.16
SNX25 + PtdIns(3,4,5)P_3	20	500	3.13 ± 0.43	-12.3 ± 0.50	-7.15	5.18	1.05 ± 0.10

**PX_Non-canonical_Mutants + PtdIns**							
SNX1_Y194A/K196A/K200A + PtdIns3*P*	20	500	NB	NB	NB	NB	NB
SNX1_Y194A/K196A/K200A + PtdIns(3,4)P_2	20	500	NB	NB	NB	NB	NB
SNX2_H187A/K189A/K193A + PtdIns3*P*	20	500	NB	NB	NB	NB	NB
SNX2_H187A/K189A/K193A + PtdIns(3,4)P_2	20	500	NB	NB	NB	NB	NB
SNX9_ Y290A/R292A/K296A + PtdIns3*P*	20	500	1.89 ± 0.38	-13.4 ± 2.08	-7.35	-7.27	0.85 ± 0.12
SNX9_Y290A/R292A/K296A + PtdIns(3,4)P_2	20	500	NB	NB	NB	NB	NB
SNX13_H620A/R622A + PtdIns(3,4)P_2	20	500	NB	NB	NB	NB	NB
SNX15_ Y64A/R67A/R71A/R72A + PtdIns3*P*	20	500	12.3 ± 0.84	-2.29 ± 0.19	-7.70	-5.41	1.21 ± 0.09
SNX15_ Y64A/R67A/R71A/R72A + PtdIns(3,4)P_2	20	500	NB	NB	NB	NB	NB
SNX23_H1224A/K1228A/K1230A + PtdIns(3,4)P_2	20	500	NB	NB	NB	NB	NB
SNX25_H558A/K560A + PtdIns(3,4,5)P_3	20	500	NB	NB	NB	NB	NB
SNX29_H315A/K317A/K321A + PtdIns3*P*	20	500	3.17 ± 0.29	-7.83 ± 0.56	-7.22	0.59	1.01 ± 0.08
SNX29_ H315A/K317A/K321A + PtdIns(3,4)P_2	20	500	NB	NB	NB	NB	NB
PI3KC2α _H1453A/K1455A + PtdIns3*P*	20	500	NB	NB	NB	NB	NB
PI3KC2α_ H1453A/K1455A + PtdIns(3,4)P_2	20	500	NB	NB	NB	NB	NB
p47phox_H51A/K55A + PtdIns3*P*	20	500	15.2 ± 2.13	-5.87 ± 1.23	-6.88	-2.74	0.93 ± 0.15
p47phox_H51A/K55A + PtdIns(3,4)P_2	20	500	NB	NB	NB	NB	NB

**Competitive titration [PX_PtdIns3*P* + PtdIns3*P*/ PtdIns(3,4)P_2]**							
SNX15_ PtdIns3*P* + PtdIns3*P*	20	500	NB	NB	NB	NB	NB
SNX15_ PtdIns3*P* + PtdIns(3,4)P_2	20	500	4.07 ±	-3.13 ± 0.17	-6.71	-5.18	0.90 ± 0.11

			0.53				
SGK3_ PtdIns3*P* + PtdIns3*P*	20	500	NB	NB	NB	NB	NB
SGK3_ PtdIns3*P* + PtdIns(3,4)P_2	20	500	9.04 ± 2.22	-4.84 ± 0.90	-6.88	-2.04	0.75 ± 0.09
SGK3_ PtdIns(3,4)P_2 + PtdIns3*P*	20	500	2.34 ± 0.51	-13.6 ± 1.07	-6.74	6.47	1.15 ± 0.11

a. Errors are standard deviation from at least two separate experiments.

Supplementary Table 5. Binding of selected PX domains to liposomes measured by BLiTz

	Conc. of PX protein (μM)	Conc. of Biotinylated Lipososome (μM)	$k_{obs.}$ ($\mu M^{-1}S^{-1}$)	K_{off} (S^{-1})	K_d (μM)
**PtdIns3*P***					
SNX1	20	500	NB	NB	NB
SNX2	20	500	NB	NB	NB
SNX3	20	500	0.0233	0.0247	1.06
SNX4	20	500	0.0121	0.0932	7.703
SNX5	20	500	NB	NB	NB
SNX6	20	500	NB	NB	NB
SNX7	20	500	0.0031	0.0098	3.161
SNX8	Not Tested	Not Tested	Not Tested	Not Tested	Not Tested
SNX9	20	500	0.0256	0.0298	1.164
SNX10	20	500	0.0236	0.3824	16.20
SNX11	20	500	0.0332	0.0305	0.9186
SNX12	20	500	0.0263	0.1901	7.228
SNX13	20	500	0.0161	0.0932	5.788
SNX14	20	500	NB	NB	NB
SNX15	20	500	0.0868	0.2733	3.1486
SNX16	20	500	0.0168	0.0932	5.54
SNX17	20	500	0.0342	0.0932	2.725
SNX18	Not Tested	Not Tested	Not Tested	Not Tested	Not Tested
SNX19	20	500	0.0121	0.0252	2.082
SNX20	Not Tested	Not Tested	Not Tested	Not Tested	Not Tested
SNX21	Not Tested	Not Tested	Not Tested	Not Tested	Not Tested
SNX22	20	500	0.0727	0.0480	0.660
SNX23	20	500	0.0599	0.0136	0.227
SNX24	20	500	0.0376	0.0235	0.625
SNX25	20	500	NB	NB	NB
SNX26	Not Tested	Not Tested	Not Tested	Not Tested	Not Tested
SNX27	20	500	0.0153	0.0387	2.529
SNX28	Not Tested	Not Tested	Not Tested	Not Tested	Not Tested
SNX29	20	500	0.0201	0.0160	0.796
SNX30	Not Tested	Not Tested	Not Tested	Not Tested	Not Tested
SNX31	20	500	0.0208	0.2097	10.08
SNX32	20	500	NB	NB	NB
SNX33	Not Tested	Not Tested	Not Tested	Not Tested	Not Tested
SNX34	Not Tested	Not Tested	Not Tested	Not Tested	Not Tested
p40phox	20	500	0.0095	0.0171	1.8
p47phox	20	500	0.0325	0.0932	2.867
PLD1	Not Tested	Not Tested	Not Tested	Not Tested	Not Tested
PLD2	Not Tested	Not Tested	Not Tested	Not Tested	Not Tested
HS1BP3	20	500	0.0256	0.0485	1.895
PXK	20	500	0.0658	0.0932	1.416
IRAS	20	500	0.0506	0.0509	1.005
SGK3	20	500	0.0217	0.0160	0.7373
SH3PXD2A	20	500	0.0154	0.1938	12.584
SH3PXD2B	Not Tested	Not Tested	Not Tested	Not Tested	Not Tested
PI3KC2α	20	500	NB	NB	NB
PI3KC2β	20	500	NB	NB	NB
PI3KC2γ	20	500	NB	NB	NB
RICS	20	500	0.0150	0.2097	13.98
RPS6KC1	20	500	0.0634	0.0509	0.8028

	Conc. of PX protein (μM)	Conc. of Biotinylated Liposfor (μM)	$k_{obs.}$ ($\mu M^{-1}S^{-1}$)	K_{off} (S^{-1})	K_d (μM)
**PtdIns(3,4)*P*$_2$**					
SNX1	20	500	0.0251	0.0201	0.80
SNX2	20	500	0.0584	0.0462	0.792
SNX5	20	500	NB	NB	NB
SNX6	20	500	NB	NB	NB
SNX9	20	500	0.0102	0.0343	3.363
SNX13	20	500	0.0070	0.0361	5.157
SNX15	20	500	0.2077	0.0343	0.165
SNX17	20	500	NB	NB	NB
SNX22	20	500	0.0327	0.0361	1.162
SNX23	20	500	0.0289	0.0302	1.04
SNX24	20	500	0.0236	0.0267	1.158
SNX25	20	500	0.0113	0.0746	6.602
SNX27	20	500	NB	NB	NB
SNX29	20	500	0.0230	0.0343	1.492
SNX32	20	500	NB	NB	NB
p40phox	20	500	NB	NB	NB
p47phox	20	500	0.0163	0.0475	2.914
HS1BP3	20	500	0.0148	0.0746	5.041
IRAS	20	500	0.0156	0.0343	2.198
SGK3	20	500	0.0207	0.0361	1.744
PI3KC2α	20	500	0.0166	0.0343	2.066
PI3KC2β	20	500	0.0243	0.0488	2.008
PI3KC2γ	20	500	0.0231	0.0348	1.506
RPS6KC1	20	500	0.0274	0.0343	1.252

	Conc. of PX protein (μM)	Conc. of Biotinylated Liposome (μM)	$k_{obs.}$ ($\mu M^{-1}S^{-1}$)	K_{off} (S^{-1})	K_d (μM)
**PtdIns(4,5)*P*$_2$**					
SNX1	20	500	0.0138	0.0343	2.485
SNX2	20	500	0.0120	0.0343	2.858
SNX5	20	500	NB	NB	NB
SNX6	20	500	NB	NB	NB
SNX15	20	500	0.0319	0.0343	1.075
SNX17	20	500	NB	NB	NB
SNX23	20	500	0.0150	0.0267	1.78
SNX24	20	500	0.0146	0.0343	2.349
SNX25	20	500	0.0533	0.0302	0.566
SNX27	20	500	NB	NB	NB
SNX29	20	500	0.0253	0.0475	1.877
p40phox	20	500	NB	NB	NB
p47phox	20	500	0.0320	0.3837	11.99
SGK3	20	500	0.0120	0.0343	2.858
PI3KC2α	20	500	0.0438	0.3837	8.76

	**Conc. of PX protein (µM)**	**Conc. of Biotinylated Lipososome (µM)**	**$k_{obs.}$ ($µM^{-1}S^{-1}$)**	**K_{off} (S^{-1})**	**K_d (µM)**
**PtdIns(3,4,5)P_3**					
SNX1	20	500	0.0169	0.1193	7.059
SNX2	20	500	0.0113	0.0746	6.602
SNX5	20	500	NB	NB	NB
SNX6	20	500	NB	NB	NB
SNX15	20	500	0.0140	0.0348	2.486
SNX17	20	500	NB	NB	NB
SNX23	20	500	0.0159	0.0343	2.157
SNX24	20	500	0.0135	0.0429	3.177
SNX25	20	500	0.0159	0.0198	1.245
SNX27	20	500	NB	NB	NB
SNX29	20	500	0.0113	0.0348	3.079
p40phox	20	500	NB	NB	NB
p47phox	20	500	0.0028	0.0304	10.857
SGK3	20	500	0.0148	0.0746	5.041
PI3KC2α	20	500	0.0153	0.0746	4.876
